# UBE2T-regulated H2AX monoubiquitination induces hepatocellular carcinoma radioresistance by facilitating CHK1 activation

**DOI:** 10.1186/s13046-020-01734-4

**Published:** 2020-10-21

**Authors:** Jingyuan Sun, Zhenru Zhu, Wenwen Li, Mengying Shen, Chuanhui Cao, Qingcan Sun, Zeqin Guo, Li Liu, Dehua Wu

**Affiliations:** 1 Department of Radiation Oncology, Nanfang Hospital, Southern Medical University, Guangzhou, 510515 China; 2 Hepatology Unit and Department of Infectious Diseases, Nanfang Hospital, Southern Medical University, Guangzhou, 510515 China

**Keywords:** Ubiquitin-conjugating enzyme E2T, H2AX monoubiquitination, Cell cycle arrest, Radioresistance, Hepatocellular carcinoma

## Abstract

**Background:**

Radioresistance is the major obstacle in radiation therapy (RT) for hepatocellular carcinoma (HCC). Dysregulation of DNA damage response (DDR), which includes DNA repair and cell cycle checkpoints activation, leads to radioresistance and limits radiotherapy efficacy in HCC patients. However, the underlying mechanism have not been clearly understood.

**Methods:**

We obtained 7 pairs of HCC tissues and corresponding non-tumor tissues, and UBE2T was identified as one of the most upregulated genes. The radioresistant role of UBE2T was examined by colony formation assays in vitro and xenograft tumor models in vivo. Comet assay, cell cycle flow cytometry and γH2AX foci measurement were used to investigate the mechanism by which UBE2T mediating DDR. Chromatin fractionation and immunofluorescence staining were used to assess cell cycle checkpoint kinase 1(CHK1) activation. Finally, we analyzed clinical data from HCC patients to verify the function of UBE2T.

**Results:**

Here, we found that ubiquitin-conjugating enzyme E2T (UBE2T) was upregulated in HCC tissues, and the HCC patients with higher UBE2T levels exhibited poorer outcomes. Functional studies indicated that UBE2T increased HCC radioresistance in vitro and in vivo. Mechanistically, UBE2T-RNF8, was identified as the E2-E3 pair, physically bonded with and monoubiquitinated histone variant H2AX/γH2AX upon radiation exposure. UBE2T-regulated H2AX/γH2AX monoubiquitination facilitated phosphorylation of CHK1 for activation and CHK1 release from the chromatin to cytosol for degradation. The interruption of UBE2T-mediated monoubiquitination on H2AX/γH2AX, including E2-enzyme-deficient mutation (C86A) of UBE2T and monoubiquitination-site-deficient mutation (K119/120R) of H2AX, cannot effectively activate CHK1. Moreover, genetical and pharmacological inhibition of CHK1 impaired the radioresistant role of UBE2T in HCC. Furthermore, clinical data suggested that the HCC patients with higher UBE2T levels exhibited worse response to radiotherapy.

**Conclusion:**

Our results revealed a novel role of UBE2T-mediated H2AX/γH2AX monoubiquitination on facilitating cell cycle arrest activation to provide sufficient time for radiation-induced DNA repair, thus conferring HCC radioresistance. This study indicated that disrupting UBE2T-H2AX-CHK1 pathway maybe a promising potential strategy to overcome HCC radioresistance.

**Supplementary information:**

**Supplementary information** accompanies this paper at 10.1186/s13046-020-01734-4.

## Background

Hepatocellular carcinoma (HCC) is the fourth leading cause of cancer-related death worldwide [1]. Liver section and transplantation comprise the first-line treatment strategy for patients with early-staged HCC. However, most of HCC patients are diagnosed at advanced stage and are ineligible for curative ablative therapies. Radiation therapy (RT) is emerging as a local ablative non-invasive treatment approach in patients with HCC. It has been reported that RT is a feasible and well-tolerated treatment for HCC patients, but confers limited tumor control benefits due to intrinsic and therapy-induced radioresistance in HCC cells [2–8].

The effectiveness of RT mostly relies on lethal damage to cancer cells by inducing double-strand DNA breaks (DSBs), which then activates DNA damage response (DDR) signaling pathway, which is composed of DNA damage repair and cell cycle checkpoint regulation [9, 10]. In response to ionizing radiation (IR)-induced DSBs, ataxia telangiectasia mutated/ataxia telangiectasia and Rad3-related protein (ATM/ATR) kinases are recruited to DSB lesion sites and become activated, which then induces phosphorylation of the histone variant H2AX (γH2AX), followed by monoubiquitination and polyubiquitination of H2AX/γH2AX. As a further consequence, DNA repair-related proteins gather at the damaged lesions and complete DNA repair [11, 12]. Meanwhile, CHK1/2 are activated by ATM/ATR to trigger cell cycle arrest and provide sufficient time for DNA repair [13, 14]. It is well known that dysregulation of the key factors involved in DDR causes altered radiosensitivity in cancer cells [15–17]. For example, Wu et al. revealed that H2AX was required for the recruitment of ATM to DNA damage foci and H2AX deficiency enhanced radiosensitivity [15]. Wang et al. demonstrated that MYC promoted radioresistance of nasopharyngeal carcinoma cells through transcriptional activation of CHK1 and CHK2 [17]. However, the means by which DNA repair and cell cycle control are integrated in the DDR is largely unknown. Thus, identifying the potential key DDR-regulatory factors and understanding the underlying mechanism involved could provide a theoretical basis for HCC radioresistance.

Ubiquitin-conjugating enzyme E2T (UBE2T) is an oncogene, widely reported to be upregulated in multiple types of cancers [18–21]. It is well documented that UBE2T is involved in HCC cell cycle modulation. Moreover, UBE2T was shown to display radio-sensitization effect on osteosarcoma and lung cancer cells. These reports shed light on the possibility that UBE2T might serve as a regulator of HCC radioresistance and DDR signal transduction cascade. As a member of the E2 family, which participates in conjugating ubiquitin to the substrates, UBE2T plays an important role in varied pathological processes in a E2-enzyme dependent manner. For example, UBE2T promotes breast cancer proliferation via polyubiquitinating and degrading BRCA1 [21]. UBE2T enhances DNA crosslinking-induced damage repair by monoubiquitinating FANCD2 [22, 23]. In this study, we explored its functional role in HCC radioresistance and the underlying mechanism.

Herein, we found that UBE2T plays a crucial role in HCC radioresistance by integrating DNA repair and cell cycle checkpoint activation, and facilitating DDR. The mechanistic study revealed that UBE2T increased H2AX monoubiquitination upon IR exposure, and then maintained CHK1 activation and promoted G2/M arrest, thus resulting in increased DDR and HCC radioresistance. Importantly, clinical evidence revealed that a correlation between UBE2T level with the response of HCC patients to RT. In summary, our findings contribute to the better understanding of HCC radioresistance and imply that targeting UBE2T might represent a therapeutic strategy for HCC radiation therapy in the future.

## Methods

### Cell culture and transfection

MHCC-97H, Huh7 and HEK-293 T cells were purchased from the Cell Bank of Type Culture Collection (CBTCC, Chinese Academy of Sciences, Shanghai, China). Cells were cultured in DMEM (Gibco, USA) supplemented with 10% fetal bovine serum (Gibco, USA) at 37 °C incubators with 5% CO_2_. All transfections were conducted using Lipofectamine 3000 (Invitrogen, USA) according to the manufacturer’s instruction.

### Clinical cohort

Thirty fresh HCC tissue specimens and the matched non-tumors tissues, which were obtained from at least 2 cm away from the tumor border and were proved to lack tumor cells by microscopy, were randomly collected from patients undergoing hepatectomy at the Nanfang Hospital affiliated to the Southern Medical University (Guangzhou, China). Another 133 HCC tissue specimens for IHC who had undergone surgery were collected at the same bank from January 2006 to July 2012. Each sample was histologically and clinically diagnosed at the Nanfang Hospital. All the patients were followed up for 5 years. Clinical information about the patients is described in detail in. Table S1–2. The disease-free survival (DFS) was defined as the interval between the date of diagnosis and the date of relapse, death, or the last observation point. The overall survival (OS) was defined as the interval between diagnosis and death or the last follow-up examination.

### Colony formation assay

Cells (400–20,000/well) were plated in 6-well plates and treated with different doses of IR (2–8 Gy). The cells were cultured for 14 days, then fixed in methanol, and stained with crystal violet. The colonies (with > 50 cells) visible to the eyes were counted. The plating efficiency (PE) was defined as the number of formed colonies/the number of seeded cells × 100%. The survival fraction is the colonies at one IR dose divided by the number of colonies with a correction for the PE. All related data were analyzed in GraphPad Prism 7 software, and survival curves of the clone formation assays were calculated using the single-hit multi-targeted model (y = 1-(1-exp(−k*x))ˆN).

### In vivo mice model

Nude mice (4–6 weeks old) were obtained from the Southern Medical University Animal Center. Ectopic tumors were established by subcutaneous injection of MHCC-97H cells (1 × 10^7^). Tumor volumes were calculated using a standard formula: length × width^2^/2. IR (6 Gy/day × 3 days) was administered to mice when tumor volume reached 100 mm^3^. Mice were divided into 4 groups (*n* > 8/group): control, UBE2T overexpression, control + IR and UBE2T overexpression + IR. For MK-8776 experiment, once tumor reached 300 mm^3^, mice were injected by MK-8776 (50 mg/kg; Sellect) intraperitoneally for 3 days. Tumors were monitored for 3 weeks. The study was approved by the Animal Care Committee of Southern Medical University.

### RNAi targeting sequence

SiRNAs were synthesized by GenePharma (Suzhou, China). Cells were transfected with the indicated siRNA using Lipofectamine® RNAiMAX (Invitrogen, USA) according to the manufacturer’s instructions. The sequences of siRNAs were as follows:

- UBE2T (5′-GCUGACAUAUCCUCAGAAUTT-3′),
- CHK1 (5′-GCAGUGAAGAUUGUAGAUATT-3′),
- H2AX(5′-TGCTGCGGAAGGGCCACTA-3′),
- RNF8(5′-GCTAGAGAATGAGCTCCAA-3′),
- TRIM21 (5′-GCTGCAGGAGGTGATAATT-3′),
- BMI1(5′-ATGAAGAGAAGAAGGGATT-3′),
- RING2(5′-GGCUAGAGCUUGAUAAUAATT-3′).

### Constructs

The full-length cDNA of UBE2T, H2AX, RNF8 and TRIM21 were subcloned into N-terminal pFLAG-CMV expression vector, respectively. The mutant constructs of UBE2T and H2AX were generated using KOD -Plus- Mutagenesis Kit (Toyobo, Japan). The constructs were confirmed by DNA sequencing. Lentivirus and adenovirus were purchased from GeneChem Company (Shanghai, China) and Vigene Corporation (Shandong, China).

### Immunoblotting

Cells were harvested, lysed in RIPA buffer containing protease inhibitors (MCE, Shanghai, China). Cell extracts were separated on 10% SDS-PAGE gels, transferred to PVDF membranes (Millipore), blocked with Tris-buffered saline/Tween 20 (TBST) containing 5% skim milk for 1 h at room temperature, and then probed with the indicated primary antibody at 4 °C overnight. After three washes, the membranes were incubated with a 1:5000 dilution of HRP-conjugated secondary antibodies for 1 h at room temperature. The immunoblots were detected using ECL (Thermo) according to the manufacturer’s protocol.

### Antibodies and reagents

The sources of antibodies against the following proteins were as follows: H2AX (D17A3; 7631), γH2AX (Ser139, 20E3; 9718), p-ATR (2853), p-ATM (13050), and p-CHK1 (Ser345, 133D3; 2348) from Cell Signaling Technology; UBE2T (10105–2-AP), CHK1 (25887–1-AP), RNF8 (14112–1-AP), TRIM21 (12108–1-AP), BRCA1 (22362–1-AP), RAD51 (14961–1-AP), BMI1 (10832–1-AP), RING2 (16031–1-AP), ATR (19787–1-AP), ATM (27156–1-AP), HSP70 (10995–1-AP) and LaminB1 (12987–1-AP) from Proteintech Group; H2AX (ab229914) from Abcam; FLAG (F1804) from Sigma-Aldrich; GAPDH (RM2002), HA (RM1004), myc (RM1003) and β-actin (RM2001) from Rui Antibody Biotech. MK-8776 (S2735) was purchased from Sellect. Cycloheximide was purchased from Sigma-Aldrich.

### Immunoprecipitation, silver staining and mass spectrometry

Lysates from the cells transfected with indicated plasmids were prepared by incubating the cells in lysis buffer containing protease inhibitor cocktail and phosphatase inhibitor (MCE, Shanghai, China). The protein was immunoprecipitated with primary antibody and the protein complexes were eluted from agarose beads. The elutes were collected and visualized on 12% Tris-Glycine SDS gel, followed by silver staining using a silver staining kit (Thermo Fisher Scientific, USA). The distinct protein bands were retrieved and analyzed by LC-MS/MS.

### Comet assay

DSB repair was analyzed by a single-cell gel electrophoresis assay using the Trevigen’s Comet Assay kit (4250–050-K) according to the manufacturer’s instructions. Briefly, cells were collected at the indicated timepoints after 4 Gy IR, immobilized in a bed of low-melting-point agarose on the CometSlides. Cells were lysed, and the remaining nucleoids were subjected to electrophoresis and subsequent staining with SYBR Gold. The presence of comet tails was examined with Olympus BX63 fluorescence microscope. Tail moment was calculated by using CASP software.

### Immunofluorescence staining analysis

Cells were fixed with 4% paraformaldehyde in phosphate-buffered saline (PBS) for 20 min, and then permeabilized in 0.5% Triton X-100 with 5% BSA in PBS for 20 min. The fixed cells were incubated overnight at 4 °C with primary antibodies against UBE2T, γH2AX, CHK1, RNF8, myc and FLAG, and then with fluorescein isothiocyanate-conjugated goat anti-mouse IgG or Cy3-conjugated goat anti-rabbit IgG (1:100) (Bioworld, China), and mounted with DAPI. An Olympus BX63 fluorescence microscope or a Carl Zeiss LSM880 confocal microscope was used for visualization.

### Cell cycle FACS analysis

The cells were washed with PBS and fixed by 70% ethanol at − 20 °C. Then, the cells were washed and incubated for 15 min at 37 °C in propidium iodide. The stained cells were analyzed using cytofluorimeter ATC300 (Bruker, France) equipped with an argon laser tuned at 488 nm.

### Chromatin fractionation

Cells were washed in PBS, and resuspended in Buffer A (10 mM Hepes pH 7.9, 10 mM KCl, 1.5 mM MgCl_2_, 0.34 M sucrose, 10% glycerol, 5 mM NaF, 1 mM Na_3_VO_4_, 1 mM DTT, and protease inhibitor mixture) containing 0.1% Triton X-100, and incubated on ice for 5 min for permeabilization. The cytosolic fraction was then separated by centrifugation at 4000 rpm for 5 min at 4 °C. The supernatant was discarded, and the nuclei pellet was washed once with Buffer A, and resuspended in Buffer B (3 mM EDTA, 0.2 mM EGTA, 1 mM DTT, protease inhibitor mixture), and incubated for 30 min on ice. The soluble nuclear fraction was separated by centrifugation at 4500 rpm for 5 min. The chromatin fraction pellet was washed with Buffer B and resuspended in 100 μL sample buffer and sonicated for 10 s before analysis.

### Immunohistochemistry staining

Immunohistochemistry (IHC) staining was carried out using a Dako Envision System according to the manufacturer’s protocol. The IHC-stained tissue section was scored by two pathologists blinded to the clinical parameters, respectively. The score of staining intensity was defined as following: 0 (negative), 1 (weak), 2 (medium), 3 (strong). The extent of staining was scored as 0 (0%), 1 (1–25%), 2 (26–50%), 3 (51–75%), 4 (76–100%), according to the percentages of the positive staining areas in relation to the entire carcinoma-involved area or the entire section for the normal samples. We regarded the sum of the intensity and extent scores as the final staining score (0–7) for UBE2T. For the purpose of statistical evaluation, tumors with a final staining score of ≥3 were considered to be high expression.

### Public database

Public TCGA (https://portal.gdc.cancer.gov/) data repositories for liver hepatocellular carcinoma (LIHC) (Cancer Genome Atlas Network, 2014) were used as the sources for the sample data. For the analysis of the LIHC TCGA sets, we used mRNA expression (by RNA sequencing).

### Gene set enrichment analysis (GSEA)

The GSEA assay using the TCGA cohort was performed to assess whether UBE2T expression is significantly correlated with certain predefined sets of genes. TC2 (c2.cp.kegg.v7.0.symbols.gmt) from the Molecular Signatures Database (MSigDB) was used as the reference gene set. UBE2T expression was annotated as a high- or low-UBE2T phenotype. A permutation number of 1000 was adopted. Results with a *P*-value less than 0.05 were regarded significant.

### Statistics

Experiments were repeated independently at least three times. Data are shown as mean ± standard deviation (SD). Student’s t-test and one-way ANOVA test were performed for comparing differences. Survival curves were plotted by the Kaplan-Meier method and compared by the log-rank test. The effects of variables on survival were determined by univariate and multivariate Cox proportional hazards model. *P* < 0.05 was considered significant.

## Results

### UBE2T is upregulated in HCC tissues and associated with patients’ survival

**Fig. 1:**
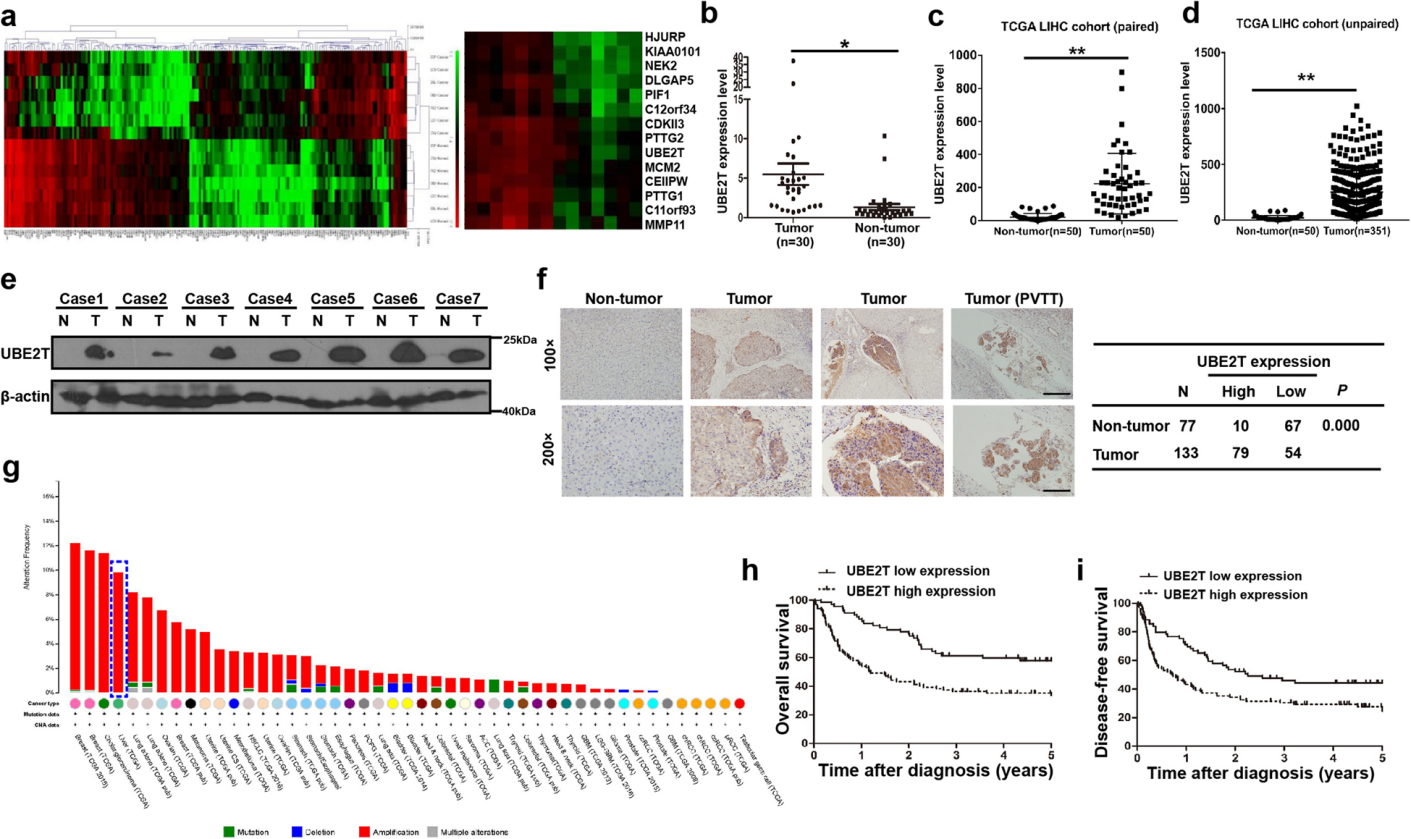
UBE2T was upregulated in HCC and was associated with survival of patients with HCC. **a** Heatmap of mRNA-Seq analysis of differentially expressed genes (two-fold change and FDR < 0.01) between specimens of 7 patients with HCC and paired non-tumor specimens (left). The upregulated genes are shown on the right. **b** UBE2T mRNA level in 30 HCC tissues was compared with that in matched non-tumor tissues. **c** UBE2T mRNA expression level in paired HCC and non-tumor tissues from the TCGA database. **d** UBE2T mRNA expression level in unpaired HCC and non-tumor tissues from the TCGA database. **e** Immunoblotting of UBE2T protein in 13 pairs of HCC tissues (T) and matched non-tumorous liver tissues (N) was performed. **f** UBE2T expression in 133 HCC tissues and 77 non-tumor tissues was analyzed by immunohistochemistry. **g** The alteration frequency of UBE2T in varied types of cancers from TCGA database at cBioPortal website. Blue frame indicates the status of UBE2T in liver cancer. **h** Kaplan-Meier survival analysis of UBE2T expression for OS in 133 patients with HCC. **i** Kaplan-Meier survival analysis of UBE2T expression for DFS in 133 HCC patients. In (**b**) and (**c**), data represent the mean ± SD. *P < 0.05, **P < 0.01, by 2-tailed paired Student’s t test. In (**d**), data represent the mean ± SD. *P < 0.05, by 2-tailed unpaired Student’s t test

We firstly identified the upregulated oncogenes in HCC tissues by performing RNA-seq on seven pairs of HCC tissues and the adjacent non-cancer tissues. We found that UBE2T was among the top upregulated genes (Fig. 1a). We then verified the RNA-seq data by investigating the mRNA level of *UBE2T* in HCC tissues and the matched non-tumor tissues from our cohort and The Cancer Genome Atlas (TCGA) public database. We found that *UBE2T* was significantly upregulated in HCC tissues compared to that in non-tumor tissues (Fig. 1b-d). The upregulation of UBE2T at the protein level in HCC tissues was also confirmed by immunoblotting (Fig. 1e). Furthermore, we analyzed UBE2T expression in 133 paraffin-embedded archival HCC tissues and 77 non-cancerous liver tissues using IHC. Results showed that of 133 HCC specimens, 79 (59.4%) showed high UBE2T expression, in comparison to only 10 (13.0%) out of 77 in the non-tumor tissue specimens (Fig. 1f). Moreover, a high rate of UBE2T amplification in liver cancer among various types of cancers was shown based on TCGA dataset from the cBioPortal website (http://www.cbioportal.org/) (Fig. 1g). Together, these findings strongly suggested that UBE2T is upregulated in HCC.

We next determined whether UBE2T expression level correlates with the outcomes for HCC patients. Results showed that patients with higher UBE2T expression had significantly shorter overall survival than those with lower UBE2T expression based on Kaplan–Meier analysis and the log-rank test (Fig. 1h). Further analysis demonstrated that patients with higher UBE2T expression levels had poorer disease-free survival (Fig. 1i). Collectively, these results suggested that UBE2T is upregulated in HCC and that high expression of UBE2T correlates with unfavorable prognosis for HCC patients.

### UBE2T promotes radioresistance and facilitates the DDR in HCC cells

**Fig. 2:**
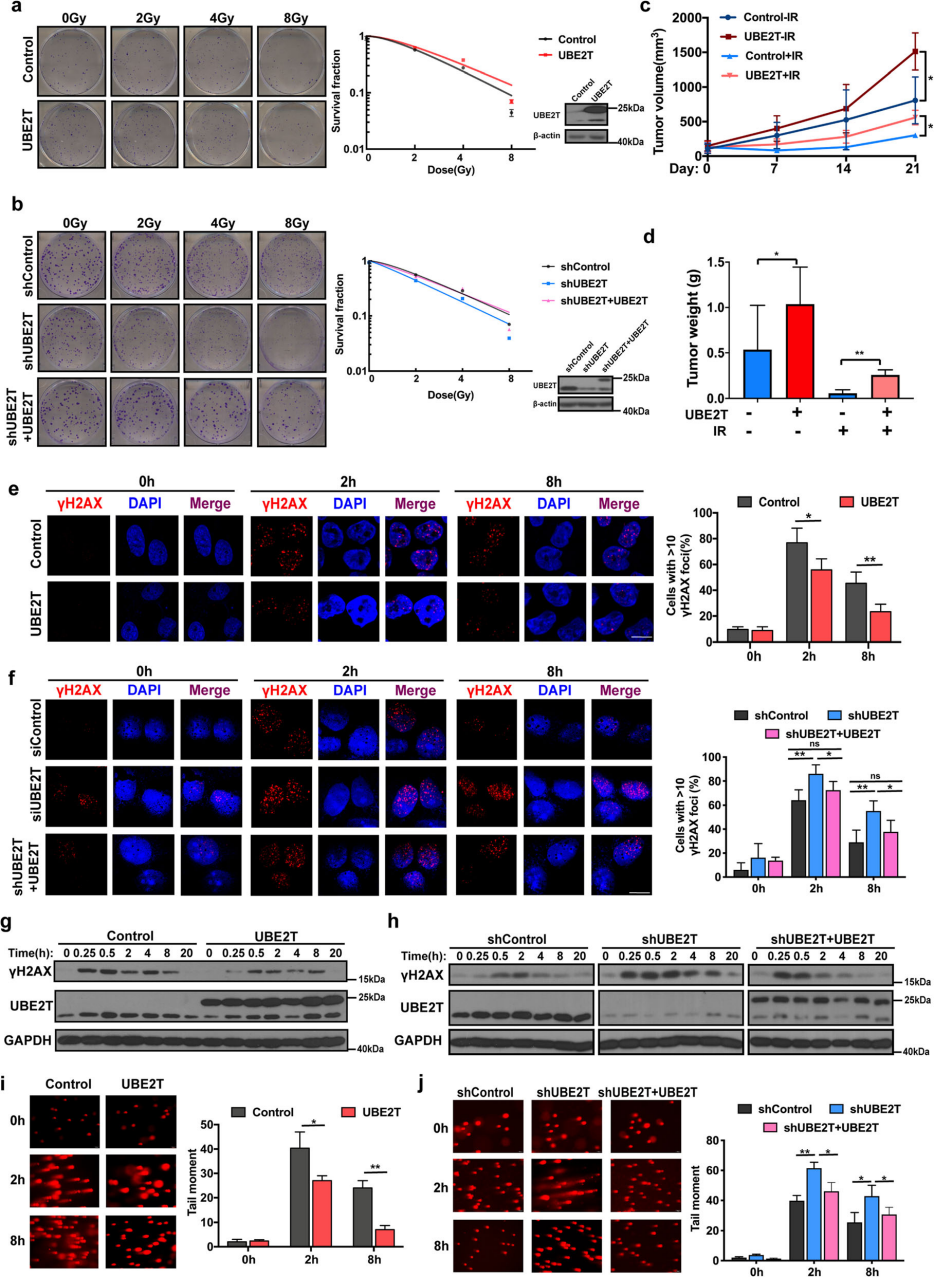
UBE2T promotes radioresistance and improves DDR of HCC after IR exposure. **a** Colony formation assays were employed in MHCC-97H cells with stable UBE2T overexpression (left, representative images of colony formation; right, survival curve and immunoblotting). **b** MHCC-97H cells were transfected with a control shRNA lentiviral vector (shControl) or shRNA lentiviral vectors targeting UBE2T (shUBE2T). Lentivirus carrying UBE2T with synonymous mutation which cannot be recognized by shUBE2T was transfected into shUBE2T MHCC-97H cells (shUBE2T + UBE2T). Colony formation assays were conducted. **c** Transplanted xenografts were established with UBE2T overexpressing (UBE2T) and vector transduced (Control) MHCC-97H cells. Tumor volumes of the xenografts from were measured for 21 days. **d** Tumor weights of the removed xenografts from panel c were measured. **e** MHCC-97H cells stably overexpressing UBE2T and control cells were exposed to IR (4 Gy). Cells were prepared for immunofluorescence analysis of γH2AX nuclear foci. The 0 timepoint indicated no IR. Representative images and quantification are shown. Scale bar: 20 μM. **f** shControl, shUBE2T and shUBE2T + UBE2T MHCC-97H cells were treated and analyzed in the same way as that in panel e. Scale bar: 20 μM. **g** Cell lysates from the cells with the same treatment as that in panel e were made for immunoblotting of γH2AX and GAPDH. **h** Cell lysates from the cells with the same treatment as that in panel f were made for immunoblotting of γH2AX and GAPDH. **i** Comet assay was performed in UBE2T overexpressing MHCC-97H cells at the indicated timepoints after IR treatment. (left, representative images; right, bar charts indicating the average tail moment per cell). **j** Cells were treated as that in panel f and made for comet assay at the indicated timepoints after IR treatment. Data represent the mean ± SD. In (**d**), (**e**), (**f**), (**i**) and (**j**), *P < 0.05 and **P < 0.01, by one-way ANOVA. In (**c**), *P < 0.05, by two-way ANOVA

By performing GSEA analysis, we found there is a significant correlation between UBE2T levels and radiation response and DNA repair (Supplementary Fig. S1a), which suggested that UBE2T may play a regulatory role in HCC radiosensitivity. To confirm this hypothesis, we first performed the colony formation assays on stable UBE2T overexpressing or knockdown MHCC-97H cells. Compared to that in control cells, the surviving fraction of HCC cells with UBE2T overexpression was increased in response to IR, whereas UBE2T silencing diminished cell colony formation after IR (Fig. 2a-b). Similar findings were observed in the cell survival assay (Supplementary Fig. S1b-c). Moreover, the re-introduction of UBE2T attenuated the role of UBE2T knockdown after increasing HCC radiosensitivity (Fig. 2b and Supplementary Fig. S2c). Next, we investigated the effect of UBE2T on the response to radiation in a nude mouse xenograft model. After establishing subcutaneous HCC xenografts, the mice were treated with X-rays (6 Gy/day for three consecutive days). Xenografts originating from MHCC-97H cells overexpressing UBE2T were larger and heavier than those originating from control cells after radiotherapy (Fig. 2c-d). H&E staining showed less necrosis and fibrosis in UBE2T-overexpressing xenografts (Supplementary Fig. S1d). These findings indicated that UBE2T conferred radioresistance in HCC cells.

It is well known that DDR is closely correlated with radioresistance in cancer cells. Next, we explored the role of UBE2T on the DDR in HCC cells. Foci formation and protein levels of γH2AX is a dynamic reflection for DDR. We found that in UBE2T-overexpressing cells, there were fewer cells with > 10 γH2AX foci, lower γH2AX expression levels after IR, and better recovery back to the basal levels, compared to those in control cells, indicative of a stronger DDR (Fig. 2e and g, Supplementary Fig. S1e-f). Similarly, there were more γH2AX foci and higher γH2AX levels in UBE2T-knockdown cells after IR, suggestive of attenuated DDR (Fig. 2f and h, Supplementary Fig. S1g-h). Additionally, we measured DSBs by performing comet assays and found that UBE2T-overexpressing cells had shorter comet tails and that UBE2T-knockdown cells formed longer tails after IR exposure (Fig. 2i-j). Moreover, the rescue experiment further confirmed the promoting role of UBE2T on DDR (Fig. 2f, h, j and Supplementary Fig. S1f, h). Therefore, together, UBE2T promotes DDR, thus leading to radioresistance in HCC cells.

### UBE2T enhances CHK1 activation and facilitates G2/M arrest in HCC

**Fig. 3:**
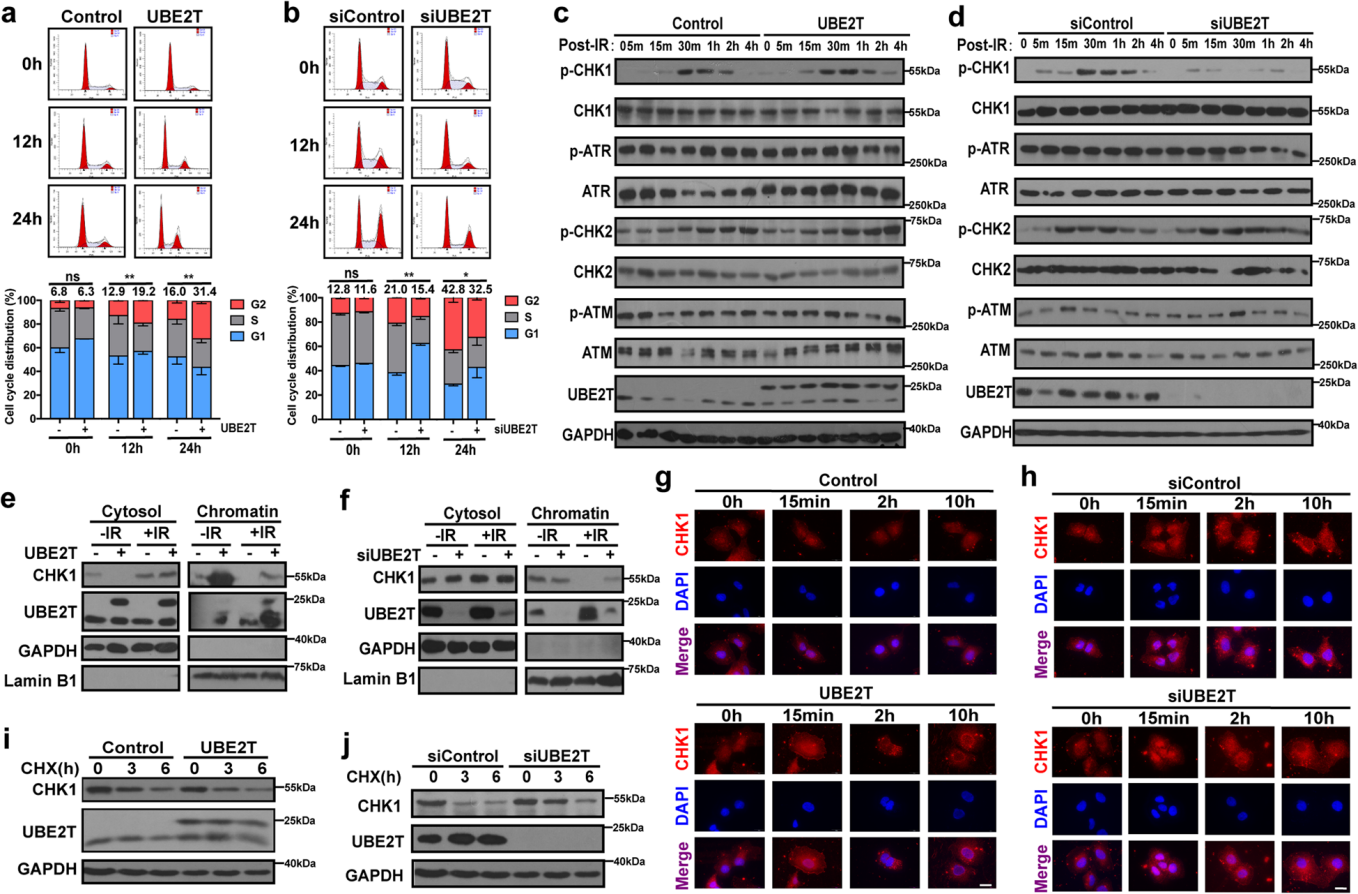
UBE2T facilitates CHK1 activation, release form chromatin to cytosol for turnover upon IR in HCC cells. **a** Cell cycle distribution was detected in MHCC-97H cells overexpressing UBE2T at indicated timepoints after IR (4 Gy). The number above the bar indicated the average percentage numbers of cells arrested in the G2 phase from triplicate experiments. **b** MHCC-97H cells with UBE2T knockdown was treated and analyzed as panel a. **c** Immunoblotting analysis of total lysates from IR (4 Gy) treated UBE2T overexpressing and control MHCC-97H cells for the indicated proteins. **d** Total protein lysates were extracted from IR (4 Gy) treated UBE2T silencing MHCC-97H cells and control cells for immunoblotting of the indicated proteins. **e** The cytosol and chromatin protein fractions of IR (4 Gy) treated UBE2T overexpressing MHCC-97H cells were isolated and analyzed. **f** The cytosol and chromatin protein fractions of IR (4 Gy) treated UBE2T silencing MHCC-97H cells were isolated and analyzed. **g** Immunofluorescence staining was used to determine the location of CHK1 in UBE2T overexpressing MHCC-97H cells at indicated timepoints after IR (4 Gy). Scale bar: 20 μM. **h** Immunofluorescent staining of CHK1 (red) in UBE2T silencing cells at indicated timepoints after IR (4 Gy). **i** UBE2T-overexpressing MHCC-97H cells were treated with IR (4 Gy) and 160 mM CHX for the indicated timepoints and immunoblotted with CHK1. **j** UBE2T-silencing MHCC-97H cells were treated with IR (4 Gy) and 160 mM CHX for the indicated timepoints and immunoblotted with CHK1. Scale bar: 20 μM. Data represent the mean ± SD. In (**a**) and (**b**), ns, not significant, *P < 0.05 and **P < 0.01, by one-way ANOVA

To uncover the mechanism underlying the effect of UBE2T on radioresistance, GSEA was utilized to identify gene sets or signatures that was correlated with UBE2T. Results revealed the enrichment of genes related to cell cycle checkpoints and G2/M checkpoints (Supplementary Fig. S2a). Thus, we first examined whether UBE2T could regulate IR-induced cell cycle alterations by performing flow cytometry on UBE2T overexpression or knockdown MHCC-97H cells together with the corresponding control cells. We found that UBE2T overexpression resulted in the accumulation of more cells in G2 phase (Fig. 3a). In contrast, UBE2T knockdown decreased the percentage of cells arrested in G2/M phase (Fig. 3b). These results indicated that UBE2T promotes IR-induced G2/M cell cycle arrest, which might contribute to DDR and radioresistance in HCC cells.

Next, we aimed to explore the factor that was modulated by UBE2T to regulate IR-induced G2/M arrest. We tested the effect of UBE2T on ATR-CHK1/ATM-CHK2 signaling pathway, and found that UBE2T overexpression dramatically enhanced the activation of CHK1 in MHCC-97H cells, shown by p-CHK1 upon IR exposure (Fig. 3c). In contrast, UBE2T knockdown impaired CHK1 activation (Fig. 3d).

It is well documented that the dissociation of CHK1 from chromatin in response to DNA damage ensures that CHK1 reaches its substrates and carries out its functions, the amount of released CHK1 strongly correlates with CHK1 activation [24, 25]. Interestingly, we found that UBE2T facilitated the interaction the association between CHK1 and ATR, meanwhile promoted CHK1 release from chromatin in MHCC-97H cells upon radiation (Fig. 3e-f, Supplementary Fig. S2b). In addition, phosphorylation at serine 345 is not only the marker of the full activation of CHK1, but also a requirement for CHK1 degradation via the proteasome pathway in the cytosol [26–29]. The turnover of CHK1 ensures that cells could continue regular cell cycle progression after IR-induced cell cycle arrest. Immunofluorescence staining for CHK1 showed that UBE2T promoted earlier cytosolic CHK1 accumulation, indicating that UBE2T promotes the translocation of CHK1 from the nucleus to the cytosol for degradation in MHCC-97H cells (Fig. 3g-h). Consistent with these findings, UBE2T caused a decrease in the stability of CHK1 in cells treated with the protein synthesis inhibitor cycloheximide after IR (Fig. 3i-j). Hence, these data suggested that UBE2T promotes CHK1 activation, chromatin release, and protein turnover in HCC cells after IR treatment.

### Genetical and pharmacological inhibition of CHK1 impairs UBE2T-induced DDR and radioresistance in HCC

**Fig. 4:**
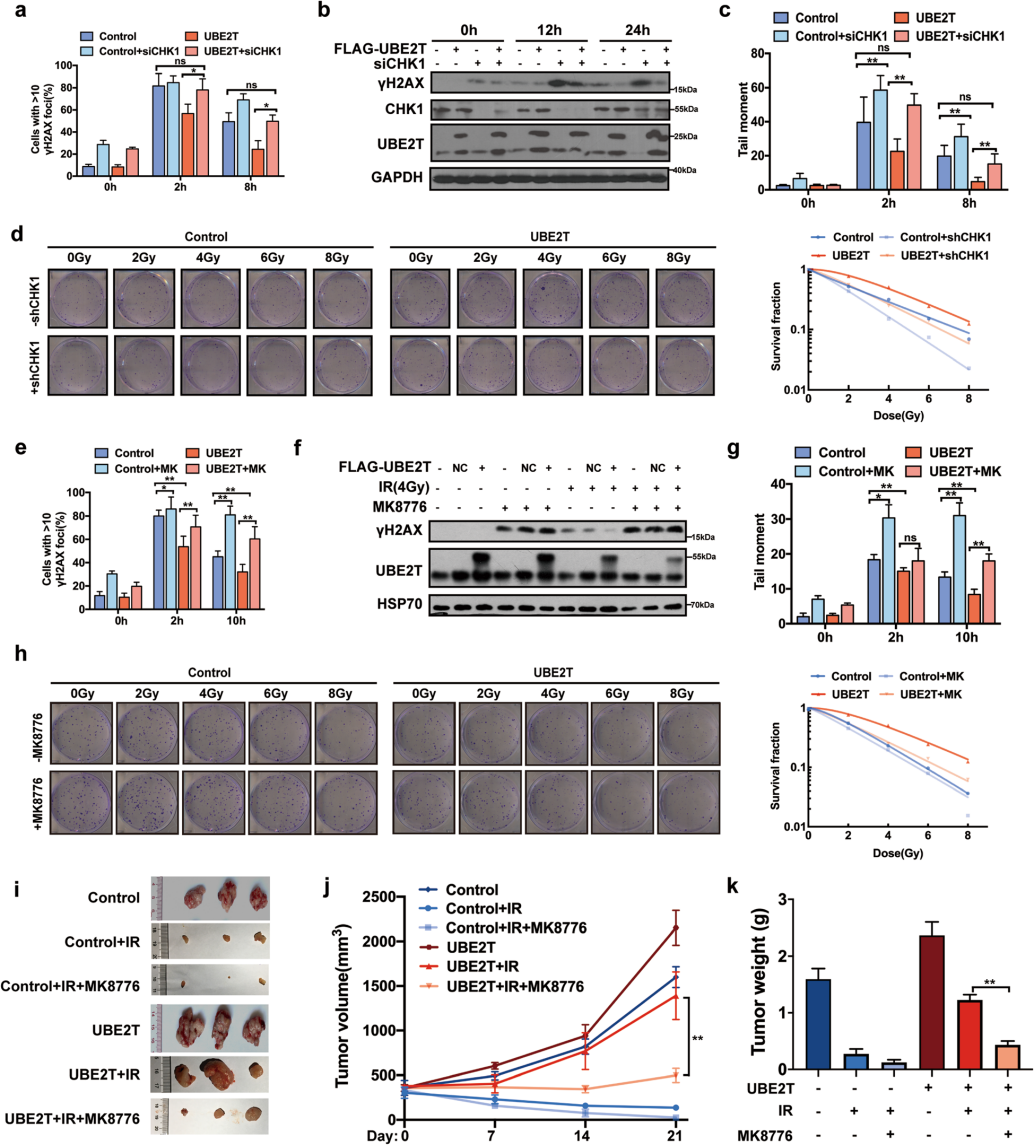
Genetical and pharmacological inhibition of CHK1 impairs UBE2T-induced DDR and radioresistance in HCC. **a** UBE2T stably overexpressing were transfected with siRNA targeting CHK1 and treated with IR (4 Gy). Cells were prepared for immunofluorescence analysis of γH2AX foci. **b** γH2AX level of lysates from cells treated as that in panel a was determined by immunoblotting. **c** Comet assay was conducted in cells with same treatment as that in panel a at indicated timepoints after IR. The quantification of the tail moment is shown. **d** Colony formation assays were conducted in UBE2T stably overexpressing MHCC-97H cells transduced with lentivirus shRNA targeting CHK1. The representative images and survival curve are shown. **e** UBE2T stably overexpression MHCC-97H cells were treated with 2 μM MK-8776 1 h before IR, and analyzed for γH2AX foci by immunofluorescence staining. **f** γH2AX level of lysates from cells treated as that in panel e was determined by immunoblotting. **g** Comet assay was conducted in cells with same treatment as that in panel e at indicated timepoints after IR. **h** Colony formation assays were conducted in UBE2T stably overexpressing MHCC-97H cells treated with 2 μM MK-8776. The representative images and survival curve are shown. **i** Transplanted xenografts were established with UBE2T overexpressing (UBE2T) and vector transduced (Control) MHCC-97H cells, and treated with IR and injected with MK-8776 (50 mg/kg) intraperitoneally for 3 days. Tumor volumes from each group were tracked for 21 days, the representative tumor samples from each group are shown. **j** Tumor volumes of the xenografts from panel i were measured. **k** Tumor weights of the removed xenografts from panel i were measured. Data represent the mean ± SD. In (**a**), (**c**), (**e**), and (**g**), ns, not significant, *P < 0.05 and **P < 0.01, by one-way ANOVA. In (**j**), **P < 0.01, by two-way ANOVA

The increased CHK1 activation level we previously observed in UBE2T-overexpressing cells suggested that inhibiting CHK1 might impair UBE2T-induced DDR and radioresistance in HCC cells. We found that knockdown of CHK1 dramatically attenuated UBE2T overexpression-induced CHK1 activation and G2/M arrest (Supplementary Fig. S2c). Meanwhile, the inhibition of CHK1 also increased γH2AX production and increased the tail length in the comet assay in UBE2T-overexpressing cells (Fig. 4a-c, Supplementary Fig. S2d-e). Furthermore, CHK1 inhibition restored radiosensitivity in UBE2T-overexpressing cell (Fig. 4d). Similar results were observed when we used CHK1 specific inhibitor MK-8776 (Fig. 4e-h, Supplementary Fig. S3). More importantly, the xenografts from UBE2T overexpressing-bearing mice treated with MK-8776 showed slower growth rate, smaller tumor volume and lighter tumor weight than those from untreated mice after radiotherapy (Fig. 4i-k). All together, these data suggested that inhibition of CHK1 impaired UBE2T-induced DDR and radioresistance in HCC.

### UBE2T facilitates CHK1 activation and G2/M arrest by binding with H2AX in HCC cells

**Fig. 5:**
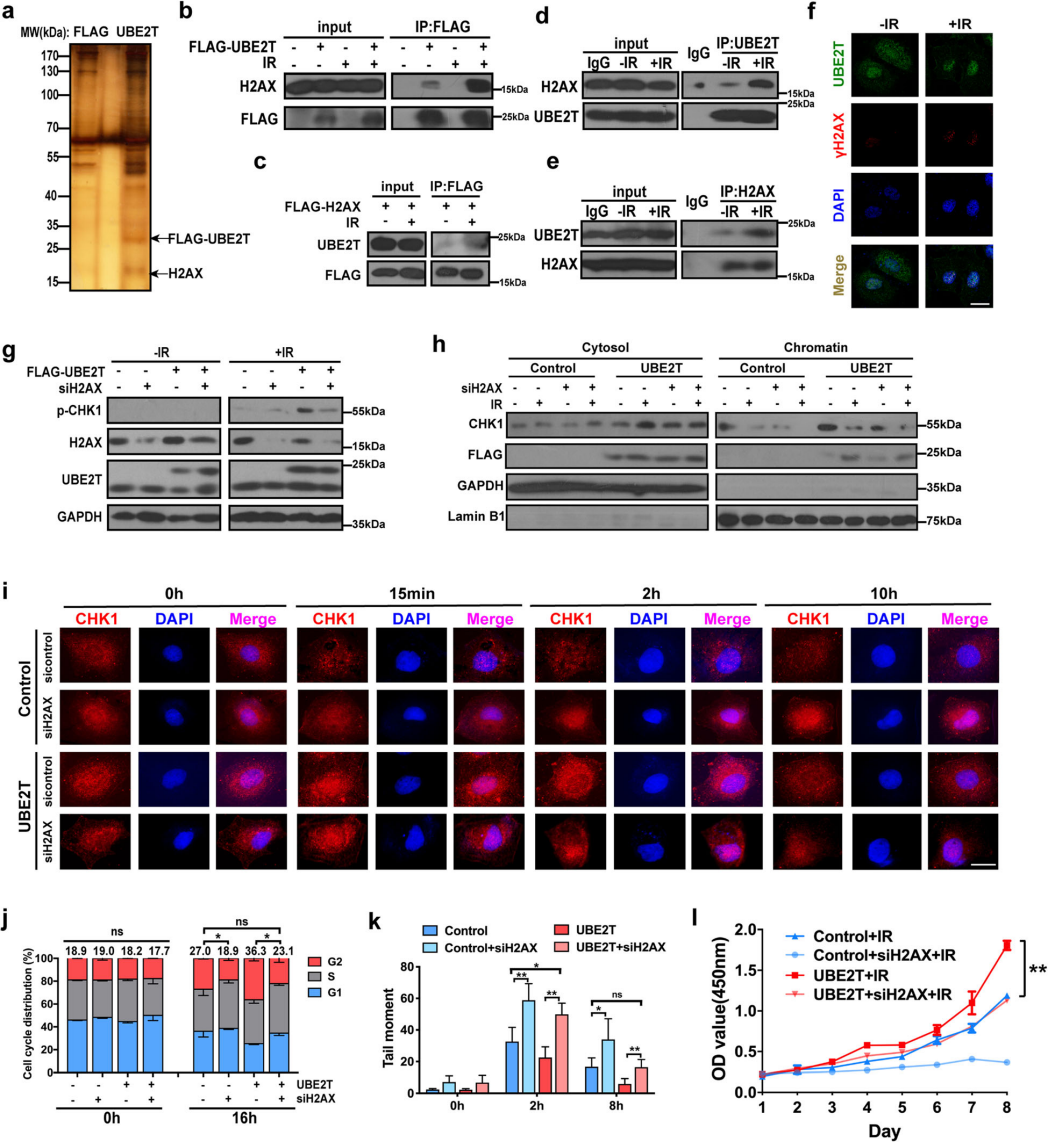
UBE2T binds with H2AX/γH2AX, and promotes CHK1 activation, DDR and HCC radioresistance via H2AX/γH2AX. **a** FLAG-UBE2T complexes were purified from 293 T cells transfected with the indicated plasmids and then treated with or without IR (4 Gy). The indicated bands were the distinct bands stained by silver stain and then identified by mass spectrometry. **b** Immunoprecipitation of H2AX with UBE2T from 293 T cells transfected with FLAG-UBE2T after IR (4 Gy). **c** Immunoprecipitation of UBE2T with H2AX from 293 T cells transfected with plasmids encoding FLAG-H2AX was analyzed by immunoblotting after IR (4 Gy). **d-e** The MHCC-97H cells treated with or without IR and made for immunoprecipitation by using anti-UBE2T antibody **(d)** or anti-H2AX antibody **(e)**. **f** MHCC-97H cells were transfected with FLAG-UBE2T and were treated with IR, FLAG-UBE2T (green), or γH2AX (red) were analyzed with immunofluorescence assay. Scale bar: 20 μM. **g** UBE2T overexpressing or control MHCC-97H cells transfected with H2AX siRNA or control siRNA, and then treated with IR (4 Gy). Total cell lysates were analyzed by immunoblotting analysis. **h** Cells were treated as panel g. The cytosol and chromatin fractionation of the cells were isolated and analyzed by immunoblotting. **i** Cells were treated as panel g. Cells were harvested at indicated timepoints, and stained for CHK1, representative images of immunofluorescence staining were shown. Scale bar: 20 μM. **j** Cells were treated as panel g. Cell cycle distribution was analyzed by flow cytometry. The average numbers of % cells in the G2 phase are shown above the bars. **k** Cells were treated as panel g. Quantification of comet assays is shown. **l** Cells were treated as panel g. Cell survival was assessed by CCK-8 assay. Data represent the mean ± SD. In (**j**) and (**k**), ns, not significant, *P < 0.05 and **P < 0.01, by one-way ANOVA. In (**l**), **P < 0.01, by two-way ANOVA

To further understand how UBE2T regulates CHK1 activation and cell cycle control, we performed a screen to identify UBE2T-binding partners by immunoprecipitation (IP) and mass spectrometric analysis upon IR exposure. Interestingly, the most striking UBE2T-interacting protein identified by was H2AX (Fig. 5a, Supplementary Fig. S4a). Bioinformatic analysis also suggested strong binding between UBE2T and H2AX (Supplementary Fig. S4b). To confirm this physical association, we performed IP and immunofluorescence staining experiments, which showed that the association between UBE2T and H2AX/γH2AX was stronger in the context of IR exposure (Fig. 5b-f and Supplementary Fig. S4c). UBE2T has a conserved core ub-conjugating (UBC) domain and an extending C-terminus [30]. Since the UBC domain is responsible for substrate recognition and E3 binding, and to determine whether the H2AX-interacting region of UBE2T is localized to the UBC domain, we constructed UBE2T vectors only harboring the UBC domain fragment. Immunoprecipitation showed that the H2AX-binding capability of the UBE2T UBC domain was slightly weaker that of full length UBE2T, and this suggested that UBE2T binds to H2AX predominantly via its UBC domain, and IR promotes this interaction (Supplementary Fig. S4d).

To determine whether the association between UBE2T and H2AX affects CHK1 activation and G2/M arrest upon IR exposure in HCC cells, we knocked down H2AX in UBE2T-overexpressing cells. We found that H2AX silencing led to lower levels of p-CHK1, less CHK1 release from chromatin, delayed CHK1 translocation from the nucleus to the cytosol, and impaired G2/M arrest after IR (Fig. 5g-j). As the following event, the knockdown of H2AX weakened UBE2T-mediated DDR and radioresistance in HCC cells (Fig. 5k-l). All these data implied that UBE2T promotes CHK1 activation and G2/M arrest via H2AX in HCC cells.

### UBE2T confers the radioresistant effect in HCC in a H2AX monoubiquitination-dependent manner

**Fig. 6:**
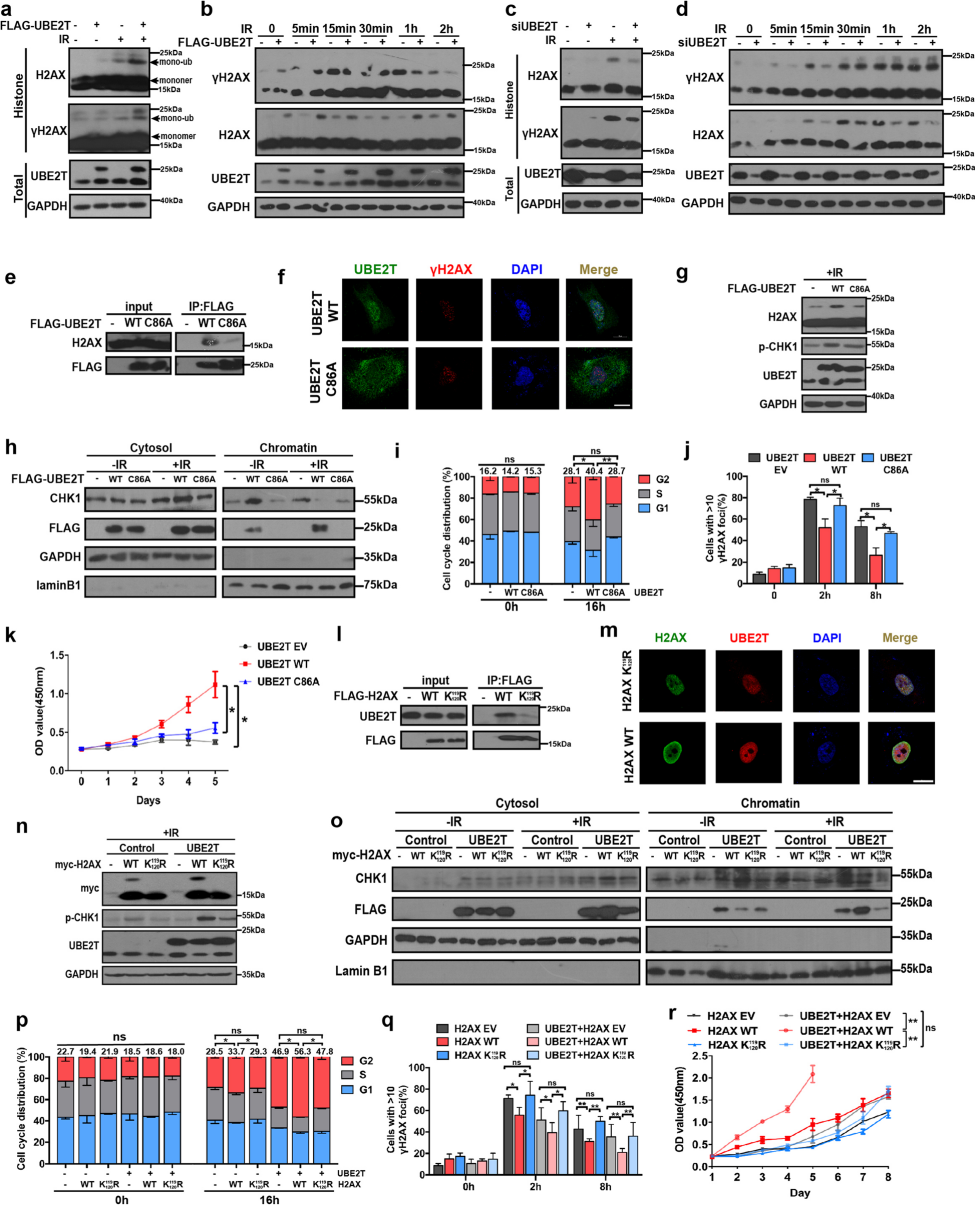
UBE2T promotes CHK1 activation, DDR and HCC radioresistance via monoubiquitinating H2AX/γH2AX. **a** Histone fraction from MHCC-97H cells transfected with FLAG-UBE2T was exposed to IR (4 Gy) and analyzed for H2AX/γH2AX monoubiquitination. **b** Immunoblotting of MHCC-97H cells expressing FLAG-UBE2T at the indicated timepoints after IR. **c** Histone fraction from MHCC-97H cells transfected with siRNA-UBE2T was exposed to IR and analyzed for H2AX/γH2AX monoubiquitination. **d** Immunoblotting of UBE2T silencing MHCC-97H cells at the indicated timepoints after IR. **e** Cell extracts from 293 T cells expressing FLAG-UBE2T WT or FLAG-UBE2T C86A were treated with IR and immunoprecipitated with anti-FLAG antibodies, followed by immunoblotting with indicated antibodies. **f-k** Cells with the same treatment as that in panel e. **f** Cells were stained for γH2AX foci (red) and FLAG-UBE2T (green). Scale bar: 20 μM. **g** Total cell lysates were analyzed by immunoblotting. **h** Cytosolic and chromatin fractions were analyzed by immunoblotting. **i** Cell cycle distribution was analyzed by flow cytometry. **j** Immunofluorescence staining was performed for γH2AX foci analysis. Quantification is shown. **k** Cell survival was assessed by CCK-8 assay. **(l)** Cellular extracts from 293 T cells expressing FLAG-H2AX WT or FLAG-H2AX K119/120R were treated with IR and immunoprecipitated with anti-FLAG antibodies, followed by immunoblotting with indicated antibodies. **m** Cells with the same treatment as that in panel l. Cells were stained for FLAG-H2AX (green) and UBE2T (red). Scale bar: 20 μM. **n-r** MHCC-97H cells were transfected with myc-tagged adenoviral-control, adenoviral-H2AX WT, adenoviral-H2AX K119/120R, or empty vector, treated with IR. **n** Total cell lysates were analyzed by immunoblotting. **o** Cytosolic and chromatin fractions were analyzed by immunoblotting. **p** Cell cycle distribution was analyzed by flow cytometry. **q** Immunofluorescence staining was performed for γH2AX foci analysis. **r** Cell survival was assessed by CCK-8 assay. Data represent the mean ± SD. In (**i**), (**j**), (**p**), and (**q**), ns, not significant, *P < 0.05 and **P < 0.01, by one-way ANOVA. In (**k**) and (**r**), ns, not significant, *P < 0.05 and **P < 0.01, by two-way ANOVA

As UBE2T is an E2 ubiquitin-conjugating enzyme and IR facilitated its interaction with H2AX/γH2AX, we hypothesized that UBE2T could regulate the ubiquitination of H2AX/γH2AX. To test this, we examined the ubiquitination of H2AX in purified histone fractions and total lysates at serial timepoints in UBE2T-overexpressing cells, and we observed an upregulated band at approximately the monoubiquitinated H2AX/γH2AX site (Fig. 6a-b). This band was further confirmed to be the monoubiquitinated H2AX/γH2AX by performing ubiquitin immunoprecipitation (Supplementary Fig. S5a). In contrast, the knockdown of UBE2T decreased H2AX/γH2AX monoubiquitination (Fig. 6c-d). In addition, E2 enzyme-deficient UBE2T (UBE2T C86A) led to a weaker association with H2AX, reduced chromatin accumulation, and less H2AX monoubiquitination (Fig. 6e-g, Supplementary Fig. S5b). Moreover, compared to that with UBE2T WT, UBE2T C86A displayed an impaired effect on CHK1 activation, which was determined based on lower p-CHK1 levels, less CHK1 release from chromatin, and later CHK1 translocation from the nucleus to the cytosol, thus leading to weaker G2/M arrest (Fig. 6h-i, Supplementary Fig. S5c). As a result, UBE2T C86A caused an impaired DDR and augmented HCC radiosensitivity (Fig. 6j-k, Supplementary Fig. S5d).

As the lysine119/120 (K119/120) residues of H2AX have been proven to be the critical monoubiquitination sites for H2AX in a previous study [15], we mutated K119/120 to arginine (H2AX K119/120R), which made H2AX unable to be monoubiquitinated. Consistent with the previous reports, we found that H2AX K119/120R rarely exhibited a monoubiquitination band and caused the persistent presence of DNA damage and weaker radioresistance in HCC cells (Supplementary Fig. S6a-b). Moreover, we found that K119/120R decreased the binding of H2AX to UBE2T, and abolished the effect of UBE2T on the upregulation of H2AX monoubiquitination ((Fig. 6l-m, Supplementary Fig. S6c). These data implied that the H2AX K119/120 site might play an essential role in UBE2T-mediated radioresistance in HCC cells. To confirm this, we transfected adenoviral-H2AX WT or adenoviral-H2AX K119/120R into UBE2T-overexpressing cells. We found that H2AX K119/120R impaired UBE2T-mediated CHK1 activation, G2/M arrest, DDR and radioresistance in HCC (Fig. 6n-r, Supplementary Fig. S7). Taken together, these results suggested that UBE2T mediates CHK1 activation, cell cycle arrest, the DDR and radioresistance, which is dependent on monoubiquitinating H2AX in HCC cells.

### UBE2T coordinates with RNF8 to enhance H2AX monoubiquitination

**Fig. 7:**
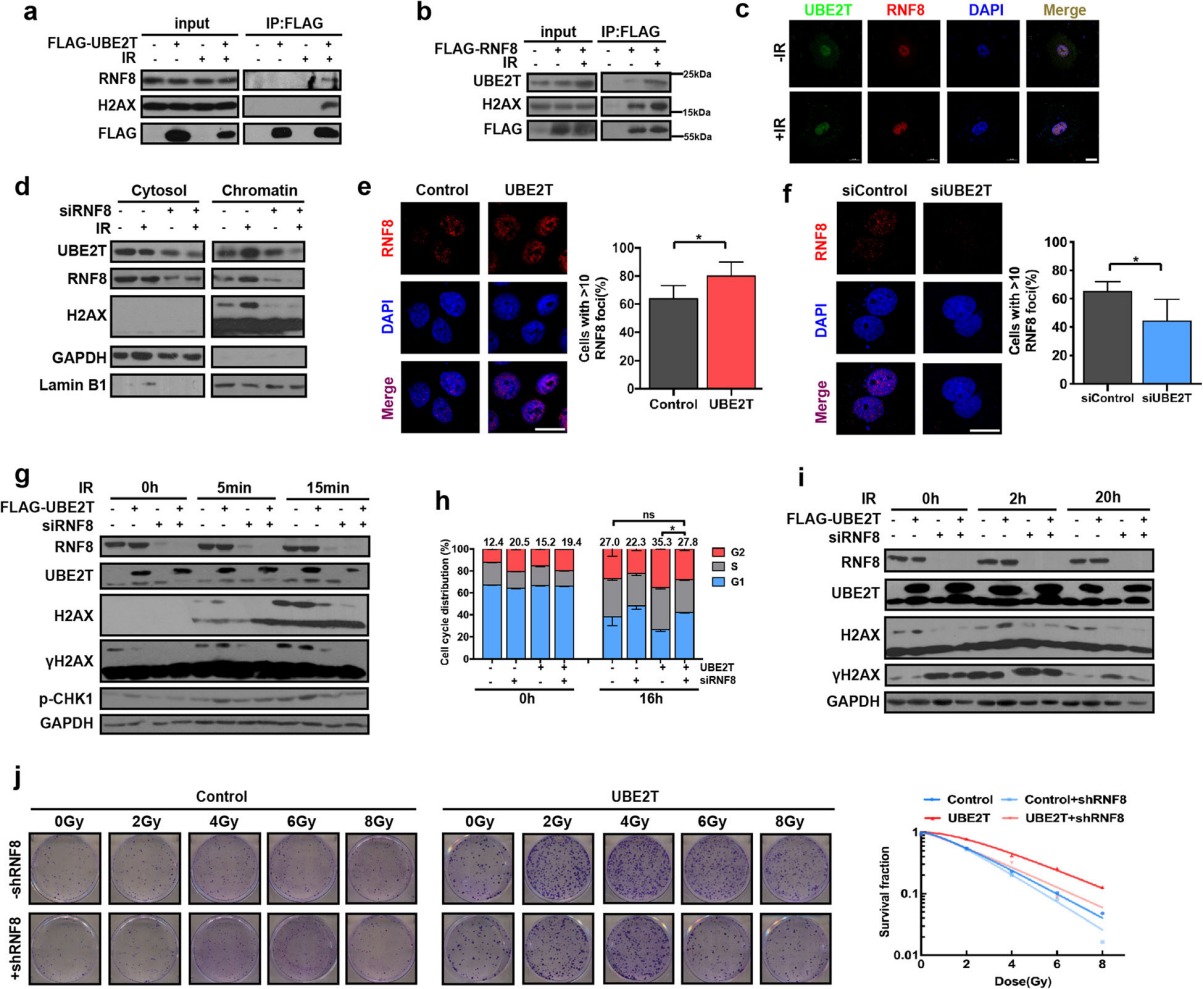
UBE2T induces HCC cell radioresistance in coordination with RNF8. **a** 293 T cells were transfected with FLAG-UBE2T and treated with IR (4 Gy). IP was performed by using FLAG antibody, and the IP product was analyzed by immunoblotting. **b** Immunoblotting analysis of FLAG-IP derived from the irradiated 293 T cells transfected with empty vector or FLAG-RNF8. **c** Representative images of immunofluorescence staining for UBE2T (green) and RNF8 (red) in MHCC-97H cells treated with IR (4 Gy). **d** Immunoblotting analysis of the cytosolic and chromatin fractions of IR (4 Gy) treated MHCC-97H cells transfected with siRNA-RNF8 or siRNA-control. **e** Representative images and quantification of UBE2T overexpressing MHCC-97H cells stained for RNF8 (red) foci before and after IR (4 Gy). **f** Representative images and quantification of UBE2T silencing MHCC-97H cells stained for RNF8 (red) foci before and after IR (4 Gy). **g** UBE2T overexpressing cells or control cells were transfected with siRNA-RNF8 or siRNA-control, and harvested at the indicated timepoints after IR (4 Gy). **h** Cells with the same treatment as that in panel g were collected at the indicated timepoints after IR (4 Gy) to test cell cycle distribution. **i** Cells with the same treatment as that in panel g were collected at the indicated timepoints after IR to test γH2AX level. **j** Colony formation assays were conducted in UBE2T stably overexpressing MHCC-97H cells transduced with lentivirus coding control shRNA or shRNA targeting RNF8. Data represent the mean ± SD. In (**e**) and (**f**), *P < 0.05, by 2-tailed paired Student’s t test. In (**h**), ns, not significant, *P < 0.05, by one-way ANOVA

It is well known that the E2-conjugating function largely relies on an E3 ligase to target the substrate. To better characterize the partners cooperating with UBE2T to monoubiquitinate H2AX/γH2AX in response to IR-induced DDR, we used IP and mass spectrometry to identify the E3 ligase that binds with UBE2T. Results indicated that two E3 ligases, TRIM21 and RNF8, were present in UBE2T-binding proteins pools (Supplementary Fig. S8a-b). IP and immunofluorescence staining after IR treatment demonstrated that UBE2T was immunoprecipitated with both TRIM21 and RNF8, and vice versa (Fig. 7a-c, Supplementary Fig. S8c-d). However, the knockdown of RNF8 but not TRIM21 diminished the effect of UBE2T on H2AX monoubiquitination and UBE2T-mediated HCC radioresistance (Supplementary Fig. S8e-g, Supplementary Fig. S9). Moreover, we found that UBE2T cannot bind with BMI1 or RING2, which are two well studied E3 ligases for H2AX monoubiquitination (Supplementary Fig. S10a). Knockdown of RNF8 further decreased H2AX monoubiquitination upon knockdown of BMI1/RING2 (Supplementary Fig. S10b). Therefore, we focused on RNF8 in the following study.

Notably, we found that blocking RNF8 after IR suppressed the chromatin binding of UBE2T, suggesting a bridging role for RNF8 between UBE2T and H2AX (Fig. 7d). Moreover, UBE2T overexpression led to an increase in the percentage of cells with > 10 RNF8 foci, and the knockdown of UBE2T decreased RNF8 foci formation in the nuclei of HCC cells after IR, indicating that UBE2T facilitates RNF8 accumulation around DSB sites in response to IR (Fig. 7e-f). More importantly, the knockdown of RNF8 abolished the effect of UBE2T on H2AX monoubiquitination and G2/M arrest, thus abolishing the radioresistance in UBE2T-overexpressing cells (Fig. 7g-j). Together, these data demonstrated that UBE2T exerts its effect on H2AX monoubiquitination, IR-induced DDR, and radioresistance in HCC cells by coordinating with RNF8.

### UBE2T levels are correlated with the local response to RT in HCC patients

**Fig. 8:**
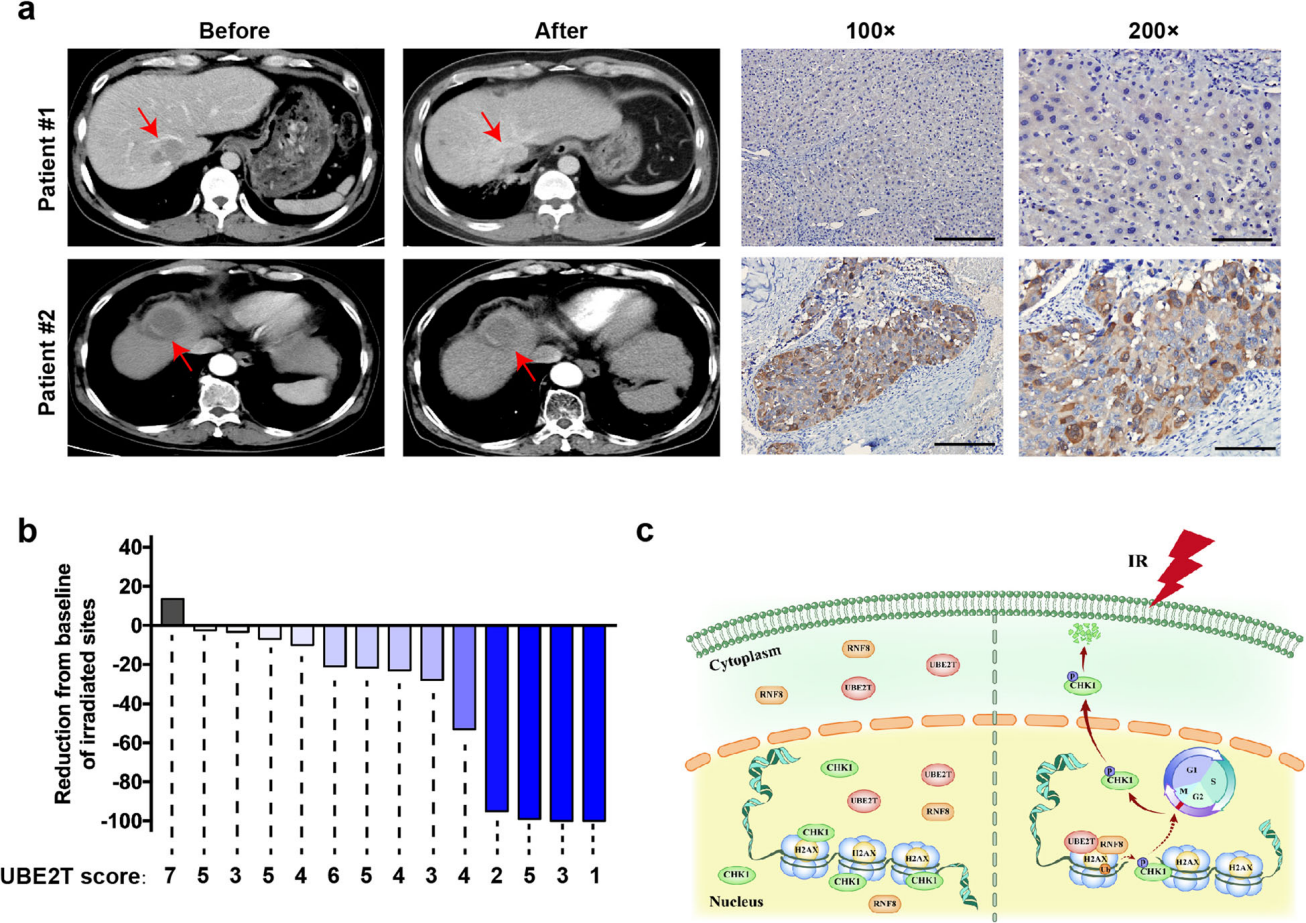
UBE2T confers resistance to RT in HCC patients. **a** CT scan images of a patient with high UBE2T level (upper panel) and a patient with low UBE2T level (lower panel), before and after RT. Red arrows indicate HCC lesions. Representative images of IHC staining for UBE2T were shown. **b** Tumor burden change from baseline in target lesions in 14 HCC patients received RT. The score of UBE2T in each patient was indicated. **c** The Working model summarizes the presented findings

Our previous data showed that UBE2T is an HCC radioresistant factor based on cellular and animal experiments. To further determine whether UBE2T has consistent effects on RT in clinical HCC patients, we collected 14 HCC specimens from patients who underwent hepatectomy or ultrasonically guided liver biopsy before RT. All patients received RT to metastatic sites localized to the liver, lung, soft tissue or lymph nodes, and all sites were assessed by CT before and after RT according to RECIST1.1 standard. Thus, we investigated UBE2T expression levels in pre-RT biopsy tumor specimens and analyzed the relationship between UBE2T levels and the radiosensitivity of metastatic sites. According to our results, UBE2T levels were lower in tumor tissues from patients exhibiting a complete response than in those from patients with local progression (Fig. 8a-b, Supplementary Table. S2). However, due to the small sample size, we were not able to perform statistical analysis. Nevertheless, our findings implied the role of UBE2T in radioresistance in HCC patients.

## Discussion

Our study suggested a radioresistant function for UBE2T in promoting the DDR and radioresistance in HCC cells. This occurs by UBE2T regulating IR-induced H2AX monoubiquitination, leading to the activation of CHK1 and cell cycle arrest (Fig. 8c). It is well demonstrated that a finely coordinated DNA repair and activation of cell cycle checkpoints, which are the two major components of DDR has evolved to cope with IR-induced DSBs. Abnormal activation of DDR has been reported to be responsible for radioresistance in cancer cells. As for the signaling transduction of DDR, ATM and ATR are well known to have crucial roles in responding to and passaging signaling in both DNA repair and checkpoint arrest pathways. ATM/ATR activates DNA repair by phosphorylating H2AX and recruiting DNA repair-related factors to the DSB lesions, while also activating cell cycle checkpoints by phosphorylating CHK1/CHK2 and initiating cell cycle arrest. Despite numerous studies have revealed the role of dysfunctional DNA repair and checkpoint arrest in tumor radioresistance, the crosstalk between these two pathways and the co-regulatory factors involved were largely unknown. H2AX deficiency was reported to impair the recruitment of DNA damage checkpoint protein 1 (MDC1) to DNA damage sites and G2/M cell cycle arrest, thus increasing radiosensitivity. These reports shed light on the possibility that H2AX is required for cell cycle checkpoint activation. In our study, we revealed a novel functional interaction between H2AX/γH2AX monoubiquitination and cell cycle modulation in the IR-induced DDR, and this process is regulated by UBE2T. As the subsequence, UBE2T facilitated sufficient cell cycle arrest, which provides abundant time for DNA repair, and then promotes the recovery of γH2AX to normal baseline levels.

Accumulating evidence has highlighted the function of the post-translational modification of H2AX. Besides phosphorylated H2AX (γH2AX), ubiquitinated H2AX is emerging as an important transducer of DDR signaling [31–33]. Increasing studies have shown that the monoubiquitination of H2AX plays a central role in regulating the cellular response to DSBs [34, 35]. A previous study showed that the monoubiquitination of H2AX, in the presence of DSBs, promotes DDR by facilitating the recruitment of MDC1 to DNA damage foci [15, 36]. Although it is widely known that increased DDR is closely associated with intrinsic radioresistance in cancer cells, there is very little data indicating a role for H2AX monoubiquitination in the radioresistance of HCC cells. As shown here, overexpressing WT H2AX but not monoubiquitination-deficient K119/120R H2AX protected HCC cells from IR in response to DNA damage, compared to that in control cells, which indicated that H2AX monoubiquitination plays an essential role in facilitating DNA damage signal passage and inducing radioresistance in HCC cells. Similar with our data, Wu et al. reported that the restoration of H2AX WT in H2AX^−/−^ mouse embryonic fibroblasts causes the resistance of the cells to IR compared to that mock restoration, whereas a K119/120R H2AX mutation compromised this effect [15]. Although this study used a non-mammalian cell system, it provided a rationale for the hypothesis that H2AX monoubiquitination has a critical role in IR sensitivity, and in our study, for the first time, we confirmed this in HCC cells.

Recent studies have illustrated the diversity of factors that regulate H2AX monoubiquitination [15, 36–38]. Among them, BMI1 and RING2, which form an E3 ligase complex, were identified as required for the accumulation of monoubiquitylated γH2AX, which is a prerequisite for γH2AX formation [15]. The other major E3 ubiquitin ligase, RNF8, was also reported to be responsible for H2AX monoubiquitination [37, 38]. In our study, we found that the knockdown of RNF8 resulted in a dramatic decrease in H2AX/γH2AX monoubiquitination. However, it should be noted that some earlier reports revealed that the knockdown of BMI1 led to a further reduction in H2AX monoubiquitination in RNF8 KO cells [39]. While in our study, we showed that UBE2T bound with RNF8, but not BMI1/RING2, and knockdown of RNF8 further decreased H2AX monoubiquitination upon knockdown of BMI1/RING2. These data suggested that RNF8, BMI1 and RING2 are all E3 ligase candidates responsible for H2AX monoubiquitination, but they may exert their roles by binding with different E2-conjugating enzymes.

Our study revealed a novel E2-conjugating enzyme, UBE2T, which interacts with RNF8 to monoubiquitinate H2AX/γH2AX. Several reports have showed that RNF8 can interact with different E2 conjugating enzymes in response to DNA damage, exerting different responses. For example, Ubc13-RNF8/RNF168 forms a K63-linked polyubiquitination chain on H2AX to recruit 53BP1 and BRCA1 to the damage sites [40, 41]. UBE2C/UBE2S-RNF8 is responsible for Lys11-linkage ubiquitin modification, which plays a role in regulating DNA damage-induced transcription inhibition [42]. This reflects the intrinsic property of E2 enzymes in synthesizing specific ubiquitin chain that executed different functional roles. While in our case, the novel interaction between UBE2T-RNF8 activates CHK1 and promotes G2/M arrest after IR via H2AX monoubiquitination. This observation was consistent with a previous report that RNF8-depleted cells failed to properly arrest at the G2/M checkpoint upon IR, and H2AX^−/−^ cells had a partially impaired G2/M checkpoint.

UBE2T was a widely reported oncogene in many types of cancers. Recently, there were several studies demonstrating the radioresistant role of UBE2T. For example, Shen et al. showed that knockdown of UBE2T has radiosensitizing role in osteosarcoma cells without exploring the underlying mechanism [20]. Yin et al. reported that UBE2T promoted radioresistance and FOXO1 ubiquitination, and FOXO1 reversed radiation resistance in lung cancer cells. (43) However, it was not illustrated that whether UBE2T conferred the radioresistant effect via ubiquitinating FOXO1. While in our study, we demonstrated that UBE2T induced DDR and HCC radioresistance by monoubiquitinating H2AX/γH2AX. Moreover, UBE2T C86A (unable to monoubiquitinate) or H2AX K119/120R (unable to be monoubiquitinated) impaired the radioresistant efficacy of UBE2T. Inconsistent with their findings that UBE2T facilitated G2/M transition, we found that UBE2T led to stronger G2/M arrest upon IR. Although such discrepancy remains unclear, one possible explanation for it may be due to the distinct cancer types we focused.

In our cohort, UBE2T was upregulated in HCC patients and could be used as a prognostic factor, and patients with higher UBE2T levels showed worse responses to RT. Thus, UBE2T could serve as a predictive biomarker for RT response to stratify patients. Due to the significant development of new techniques, RT has emerged as an effective treatment for HCC patients at almost every stage. However, surgery remains the first option for patients with early stage HCC. In our cohort, most of the patients were diagnosed in the early stages of HCC and underwent surgery, followed by receiving RT at metastatic sites. However, we cannot exclude the possibility of heterogeneous alterations during the metastatic process. Collectively, our findings might provide new insights for understanding the DDR and radioresistance in HCC cells, and could contribute to the development of precise treatment strategies for clinical practice in the future. Future study may further screen or develop novel compounds to target UBE2T signaling pathway, which may be a new adjuvant therapy for RT in HCC.

## Conclusion

In summary, we found that UBE2T maintained CHK1 activation and promoted G2/M arrest by increasing H2AX monoubiquitination upon IR exposure. Our findings identified UBE2T/RNF8-H2AX-CHK1 as a novel pathway of DDR and HCC radioresistance, highlighting the prospect and merit of disrupting this pathway to develop adjuvant treatments for HCC RT. Importantly, clinical evidence revealed a correlation between UBE2T levels and the response of HCC patients to RT. In summary, our findings contribute to a better understanding of HCC radioresistance, and imply that targeting UBE2T might represent a therapeutic strategy for HCC RT in the future.

## Supplementary information

**Additional file 1: Fig. S1.** UBE2T regulates HCC radioresistance and DDR. **Fig. S2.** Genetical inhibition of CHK1 impairs UBE2T-induced G2/M arrest and DDR in HCC. **Fig. S3.** Pharmacological inhibition of CHK1 impairs UBE2T-induced G2/M arrest and DDR in HCC. **Fig. S4.** UBE2T binds with H2AX by UBC domain. **Fig. S5.** UBE2T C86A inhibits the chromatin accumulation of UBE2T, translocation of CHK1 from nuclear to cytosol and DDR after IR treatment. **Fig. S6.** K119/120 are critical monoubiquitination sites and UBE2T regulates H2AX monoubiquitination after IR treatment in HCC cells. **Fig. S7.** K119/120R impairs the role of UBE2T in promoting the translocation of CHK1 from nuclear to cytosol and DDR after IR treatment. **Fig. S8.** UBE2T regulates H2AX monoubiquitination together with RNF8, but not TRIM21, after IR in HCC cells. **Fig. S9.** Knockdown of RNF8 but not TRIM21 reverts the effect of UBE2T on DDR and radioresistance. **Fig. S10.** BMI1/RING2 don’t bind with UBE2T after IR exposure, and knockdown of RNF8 further decrease H2AX monoubiquitination upon knockdown of BMI1/RING2. **Table. S1.** Clinicopathological characteristics of 133 HCC patients. **Table. S2.** Clinicopathological characteristics of 14 HCC patients received RT.

## Data Availability

The data generated and/or analyzed during the current study are available upon reasonable request to the corresponding author. The microarray data were deposited in NCBI Gene Expression Omnibus and are accessible through GEO series accession number GSE58043.

## Abbreviations

A: Alanine
ATM: Ataxia telangiectasia mutated
ATR: Ataxia telangiectasia and Rad3-related protein
C: Cysteine
CHK1: Cell cycle checkpoint kinase 1
CHK2: Cell cycle checkpoint kinase 2
CHX: Cycloheximide
DDR: DNA damage response
DFS: Disease-free survival
DSB: Double strands break
GSEA: Gene-set enrichment analysis
HCC: Hepatocellular carcinoma
IP: Immunoprecipitation
IR: Ionizing radiation
K: Lysine
MDC1: Mediator of DNA damage checkpoint protein 1
OS: Overall survival
R: Arginine
RNF8: RING finger protein 8
TRIM21: Tripartite motif-containing protein 21
UBE2T: Ubiquitin-conjugating enzyme E2 T
WT: Wild type
BMI1: B-cell specific Moloney murine leukemia virus integration site 1
RING2: Ring finger protein 2

## References

[1] Bray F, Ferlay J, Soerjomataram I, Siegel RL, Torre LA, Jemal A (2018). Global cancer statistics 2018: GLOBOCAN estimates of incidence and mortality worldwide for 36 cancers in 185 countries. CA Cancer J Clin.

[2] Bartkova J, Horejsi Z, Koed K, Kramer A, Tort F, Zieger K (2005). DNA damage response as a candidate anti-cancer barrier in early human tumorigenesis. Nature..

[3] Curtin NJ (2012). DNA repair dysregulation from cancer driver to therapeutic target. Nat Rev Cancer.

[4] Jeggo PA, Pearl LH, Carr AM (2016). DNA repair, genome stability and cancer: a historical perspective. Nat Rev Cancer.

[5] Lord CJ, Ashworth A (2012). The DNA damage response and cancer therapy. Nature..

[6] Wahl DR, Stenmark MH, Tao Y, Pollom EL, Caoili EM, Lawrence TS (2016). Outcomes after stereotactic body radiotherapy or radiofrequency ablation for hepatocellular carcinoma. J Clin Oncol.

[7] Rajyaguru DJ, Borgert AJ, Smith AL, Thomes RM, Conway PD, Halfdanarson TR (2018). Radiofrequency ablation versus stereotactic body radiotherapy for localized hepatocellular carcinoma in nonsurgically managed patients: analysis of the National Cancer Database. J Clin Oncol.

[8] Yoon HI, Seong J (2014). Multimodality treatment involving radiotherapy for advanced liver-confined hepatocellular carcinoma. Oncology..

[9] Datta K, Jaruga P, Dizdaroglu M, Neumann RD, Winters TA (2006). Molecular analysis of base damage clustering associated with a site-specific radiation-induced DNA double-strand break. Radiat Res.

[10] Lord CJ, Garrett MD, Ashworth A (2006). Targeting the double-strand DNA break repair pathway as a therapeutic strategy. Clin Cancer Res.

[11] Price BD, D'Andrea AD (2013). Chromatin remodeling at DNA double-strand breaks. Cell..

[12] Xu Y, Price BD (2011). Chromatin dynamics and the repair of DNA double strand breaks. Cell Cycle.

[13] Smith J, Tho LM, Xu N, Gillespie DA (2010). The ATM-Chk2 and ATR-Chk1 pathways in DNA damage signaling and cancer. Adv Cancer Res.

[14] Smits VA, Gillespie DA (2015). DNA damage control: regulation and functions of checkpoint kinase 1. FEBS J.

[15] Wu CY, Kang HY, Yang WL, Wu J, Jeong YS, Wang J (2011). Critical role of monoubiquitination of histone H2AX protein in histone H2AX phosphorylation and DNA damage response. J Biol Chem.

[16] Bassing CH, Alt FW (2004). H2AX may function as an anchor to hold broken chromosomal DNA ends in close proximity. Cell Cycle.

[17] Wang WJ, Wu SP, Liu JB, Shi YS, Huang X, Zhang QB (2013). MYC regulation of CHK1 and CHK2 promotes radioresistance in a stem cell-like population of nasopharyngeal carcinoma cells. Cancer Res.

[18] Zhang W, Zhang Y, Yang Z, Liu X, Yang P, Wang J, et al. High expression of UBE2T predicts poor prognosis and survival in multiple myeloma. Cancer Gene Ther. 2019. 10.1038/s41417-018-0070-x PMC6892417 30622320

[19] Liu LP, Yang M, Peng QZ, Li MY, Zhang YS, Guo YH (2017). UBE2T promotes hepatocellular carcinoma cell growth via ubiquitination of p53. Biochem Biophys Res Commun.

[20] Wang Y, Leng H, Chen H, Wang L, Jiang N, Huo X (2016). Knockdown of UBE2T inhibits osteosarcoma cell proliferation, migration, and invasion by suppressing the PI3K/Akt signaling pathway. Oncol Res.

[21] Ueki T, Park JH, Nishidate T, Kijima K, Hirata K, Nakamura Y (2009). Ubiquitination and downregulation of BRCA1 by ubiquitin-conjugating enzyme E2T overexpression in human breast cancer cells. Cancer Res.

[22] Che R, Zhang J, Nepal M, Han B, Fei P (2018). Multifaceted Fanconi Anemia Signaling. Trends Genet.

[23] Nepal M, Che R, Ma C, Zhang J, Fei P. FANCD2 and DNA Damage. Int J Mol Sci. 2017;18(8). 10.3390/ijms18081804 PMC5578191 28825622

[24] Smits VA (2006). Spreading the signal: dissociation of Chk1 from chromatin. Cell Cycle.

[25] Smits VA, Reaper PM, Jackson SP (2006). Rapid PIKK-dependent release of Chk1 from chromatin promotes the DNA-damage checkpoint response. Curr Biol.

[26] Yarden RI, Metsuyanim S, Pickholtz I, Shabbeer S, Tellio H, Papa MZ (2012). BRCA1-dependent Chk1 phosphorylation triggers partial chromatin disassociation of phosphorylated Chk1 and facilitates S-phase cell cycle arrest. Int J Biochem Cell Biol.

[27] Leung-Pineda V, Huh J, Piwnica-Worms H (2009). DDB1 targets Chk1 to the Cul4 E3 ligase complex in normal cycling cells and in cells experiencing replication stress. Cancer Res.

[28] Zhang YW, Brognard J, Coughlin C, You Z, Dolled-Filhart M, Aslanian A (2009). The F box protein Fbx6 regulates Chk1 stability and cellular sensitivity to replication stress. Mol Cell.

[29] Merry C, Fu K, Wang J, Yeh IJ, Zhang Y (2010). Targeting the checkpoint kinase Chk1 in cancer therapy. Cell Cycle.

[30] Hormaechea-Agulla D, Kim Y, Song MS, Song SJ (2018). New insights into the role of E2s in the pathogenesis of diseases: lessons learned from UBE2O. Mol Cell.

[31] Vidanes GM, Bonilla CY, Toczyski DP (2005). Complicated tails: histone modifications and the DNA damage response. Cell..

[32] Bonner WM, Redon CE, Dickey JS, Nakamura AJ, Sedelnikova OA, Solier S (2008). GammaH2AX and cancer. Nat Rev Cancer.

[33] Lukas J, Lukas C, Bartek J (2011). More than just a focus: the chromatin response to DNA damage and its role in genome integrity maintenance. Nat Cell Biol.

[34] So CC, Ramachandran S, Martin A. E3 Ubiquitin Ligases RNF20 and RNF40 Are Required for Double-Stranded Break (DSB) Repair: Evidence for Monoubiquitination of Histone H2B Lysine 120 as a Novel Axis of DSB Signaling and Repair. Mol Cell Biol. 2019;39(8). 10.1128/MCB.00488-18 PMC6447412 30692271

[35] Yamamoto T, Taira Nihira N, Yogosawa S, Aoki K, Takeda H, Sawasaki T (2017). Interaction between RNF8 and DYRK2 is required for the recruitment of DNA repair molecules to DNA double-strand breaks. FEBS Lett.

[36] Pan MR, Peng G, Hung WC, Lin SY (2011). Monoubiquitination of H2AX protein regulates DNA damage response signaling. J Biol Chem.

[37] Mailand N, Bekker-Jensen S, Faustrup H, Melander F, Bartek J, Lukas C (2007). RNF8 ubiquitylates histones at DNA double-strand breaks and promotes assembly of repair proteins. Cell..

[38] Huen MS, Grant R, Manke I, Minn K, Yu X, Yaffe MB (2007). RNF8 transduces the DNA-damage signal via histone ubiquitylation and checkpoint protein assembly. Cell..

[39] Ismail IH, Andrin C, McDonald D, Hendzel MJ (2010). BMI1-mediated histone ubiquitylation promotes DNA double-strand break repair. J Cell Biol.

[40] Hodge CD, Ismail IH, Edwards RA, Hura GL, Xiao AT, Tainer JA (2016). RNF8 E3 ubiquitin ligase stimulates Ubc13 E2 conjugating activity that is essential for DNA double Strand break signaling and BRCA1 tumor suppressor recruitment. J Biol Chem.

[41] Lee BL, Singh A, Mark Glover JN, Hendzel MJ, Spyracopoulos L (2017). Molecular basis for K63-linked Ubiquitination processes in double-Strand DNA break repair: a focus on kinetics and dynamics. J Mol Biol.

[42] Paul A, Wang B (2017). RNF8- and Ube2S-dependent ubiquitin lysine 11-linkage modification in response to DNA damage. Mol Cell.

