# Supplementary material for: UBE2T-regulated H2AX monoubiquitination induces hepatocellular carcinoma radioresistance by facilitating CHK1 activation
Source: J Exp Clin Cancer Res. 2020 Oct 21;39:222. doi: 10.1186/s13046-020-01734-4 (PMC7576867; doi:10.1186/s13046-020-01734-4)
Supplement: Supplementary file 1 — Additional file 1: Fig. S1. UBE2T regulates HCC radioresistance and DDR. Fig. S2. Genetical inhibition of CHK1 impairs UBE2T-induced G2/M arrest and DDR in HCC. Fig. S3. Pharmacological inhibition of CHK1 impairs UBE2T-induced G2/M arrest and DDR in HCC. Fig. S4. UBE2T binds with H2AX by UBC domain. Fig. S5. UBE2T C86A inhibits the chromatin accumulation of UBE2T, translocation of CHK1 from nuclear to cytosol and DDR after IR treatment. Fig. S6. K119/120 are critical monoubiquitination sites and UBE2T regulates H2AX monoubiquitination after IR treatment in HCC cells. Fig. S7. K119/120R impairs the role of UBE2T in promoting the translocation of CHK1 from nuclear to cytosol and DDR after IR treatment. Fig. S8. UBE2T regulates H2AX monoubiquitination together with RNF8, but not TRIM21, after IR in HCC cells. Fig. S9. Knockdown of RNF8 but not TRIM21 reverts the effect of UBE2T on DDR and radioresistance. Fig. S10. BMI1/RING2 don’t bind with UBE2T after IR exposure, and knockdown of RNF8 further decrease H2AX monoubiquitination upon knockdown of BMI1/RING2. Table. S1. Clinicopathological characteristics of 133 HCC patients. Table. S2. Clinicopathological characteristics of 14 HCC patients received RT. [file 13046_2020_1734_MOESM1_ESM.docx]

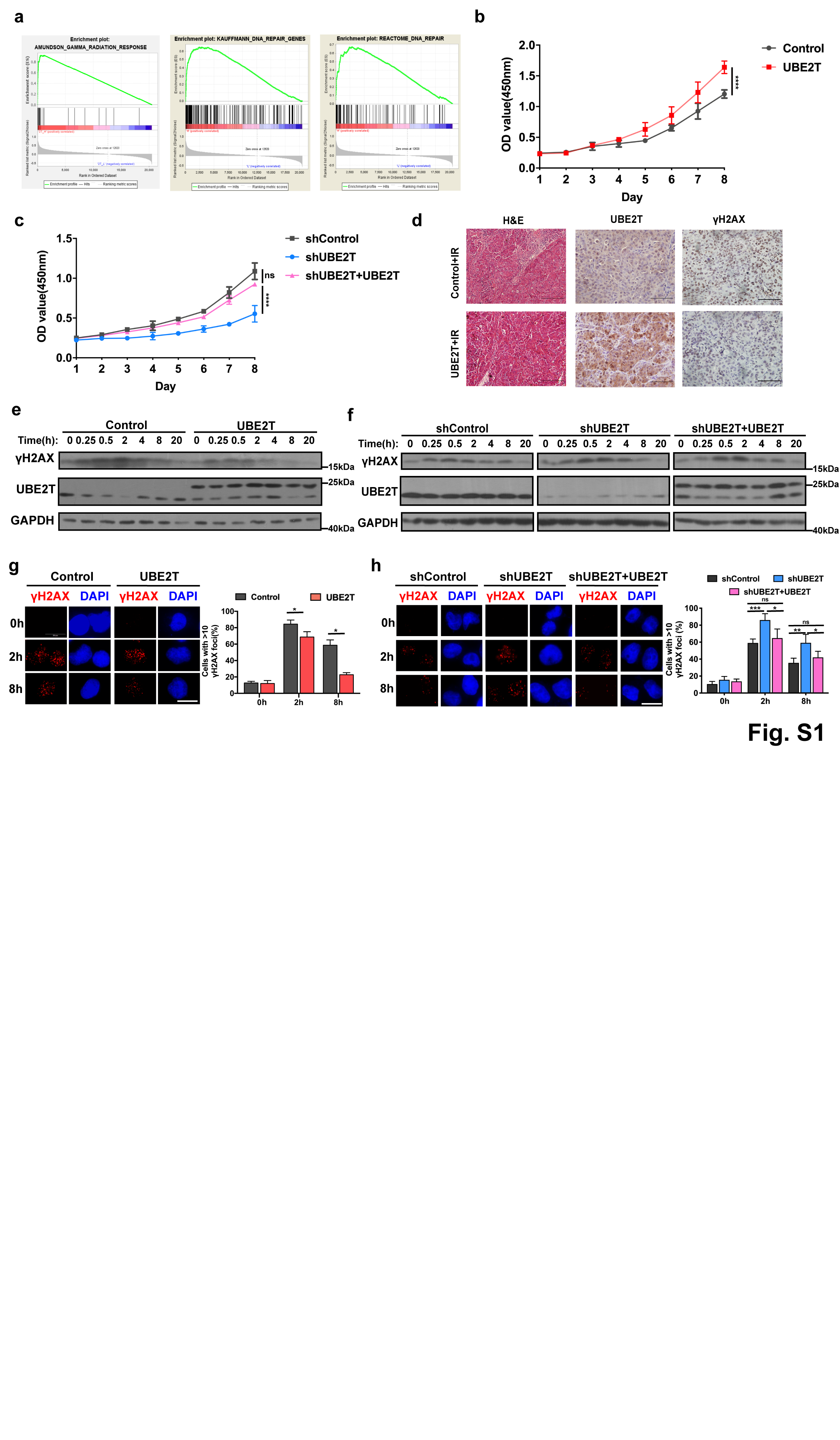


**Fig. S1 UBE2T regulates HCC radioresistance and DDR. (a)** Result of Gene set enrichment analysis (GSEA) was plotted to visualize the correlation between the expression of UBE2T and gene signatures of radiation response and DNA repair in the TCGA cohort. **(b)** Cell survival of in Huh7 cells with stable UBE2T overexpression was assessed by CCK-8 assay. **(c)** Huh7 cells were transfected with a control shRNA lentiviral vector (shControl) or shRNA lentiviral vectors targeting UBE2T (shUBE2T). Lentivirus carrying mutant UBE2T which cannot be recognized by shUBE2T was transfected into shUBE2T MHCC-97H cells (shUBE2T+UBE2T). Cell survival assays were conducted. **(d)** Representative images of H&E staining and IHC staining in the harvested xenografts from Fig. 2c. Scale bar, 100μM. **(e)** Stably overexpressing UBE2T and control Huh7 cells were collected at the indicated timepoints after IR. Cell lysates were analyzed by immunoblotting. **(f)** Cells with same treatment in panel d were made for immunofluorescence staining of γH2AX. Scale bar, 20μM. **(g)** UBE2T silencing cells and control Huh7 cells were collected at the indicated timepoints after IR. Cell lysates were analyzed by immunoblotting. **(h)** Cells with same treatment as that in panel d were made for immunofluorescence staining of γH2AX. Scale bar, 20μM. For (g) and (h), representative images and quantification of cells with > 10 foci from triplicate experiments are shown. Scale bar: 20μM. Data represent the mean ± SD. **P* < 0.05, by one-way ANOVA.


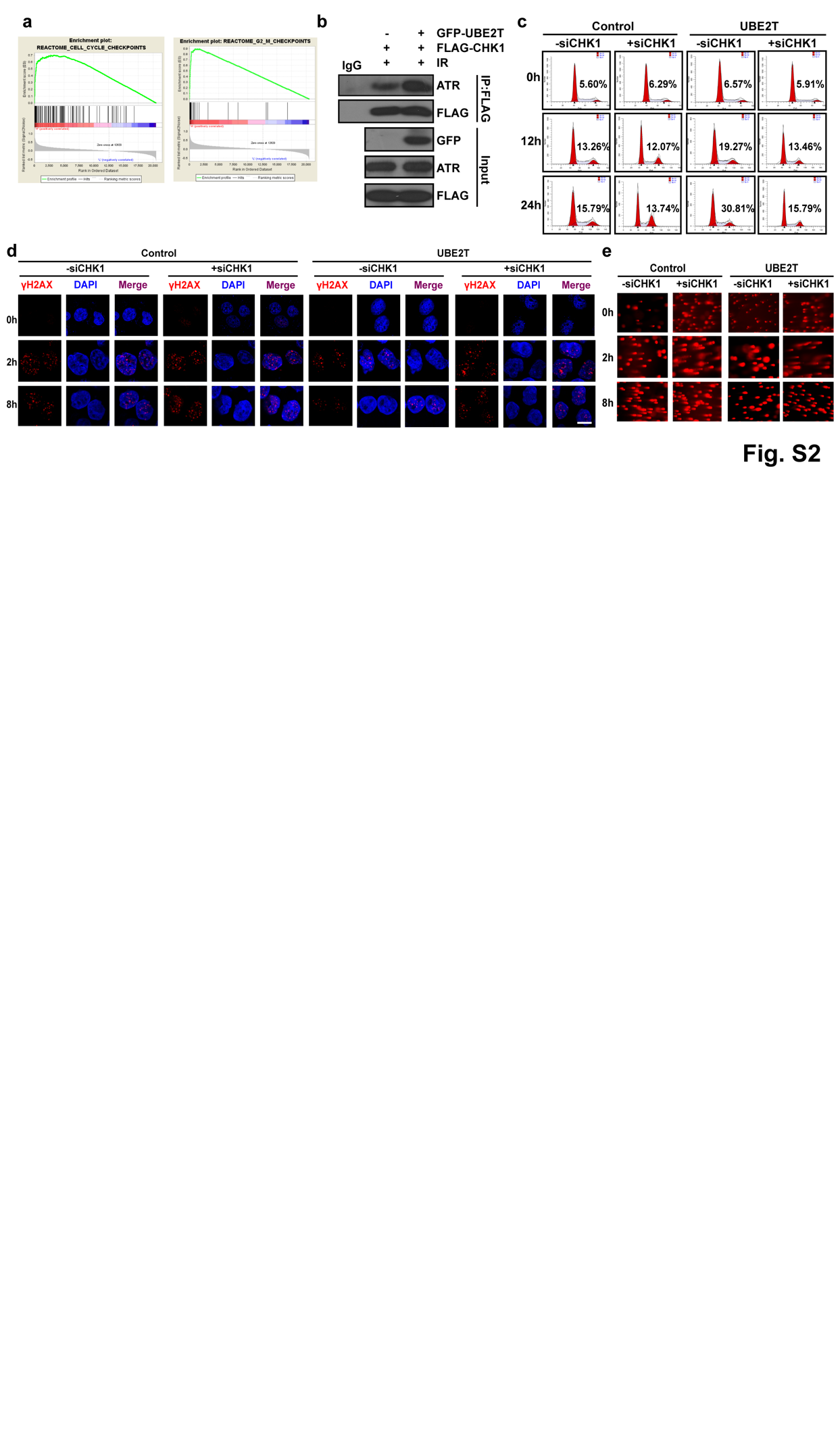


**Fig. S2 Genetical inhibition of CHK1 impairs UBE2T-induced G2/M arrest and DDR in HCC.** **(a)** Result of Gene set enrichment analysis (GSEA) was plotted to visualize the correlation between the expression of UBE2T and gene signatures of cell cycle checkpoints in the TCGA cohort. **(b)** Immunoprecipitation of CHK1 complex by using anti-FLAG antibody from 293T cells transfected with FLAG-CHK1 together with GFP-UBE2T or GFP vectors after IR (4 Gy). **(c)** UBE2T stably overexpressing MHCC-97H cells were transfected with control siRNA or siRNA targeting CHK1 and treated with IR (4 Gy). Cell cycle distribution was detected, the numbers of % cells in the G2 phase are shown. **(d)** Cells were treated as that in panel c, then were made for immunofluorescence staining of γH2AX. Representative images from triplicate experiments were shown, the quantification is shown in Fig. 4a. Scale bar, 20μM. **(e)** Cells were treated as that in panel c, and analyzed for DNA damage by comet assay. Bar chart indicating the average tail moment per cell from triplicate experiments was shown in Fig. 4c.


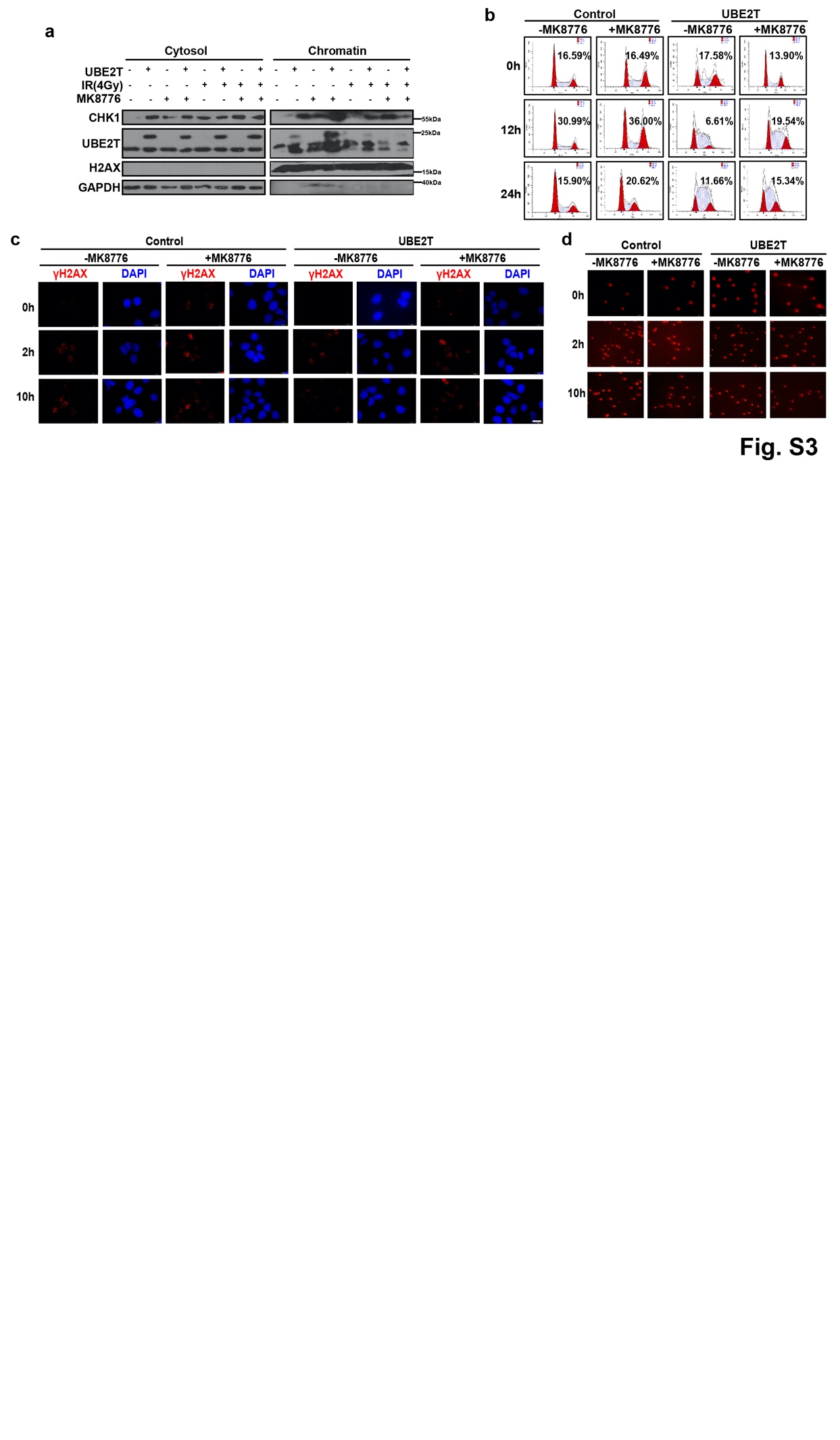


**Fig. S3 Pharmacological inhibition of CHK1 impairs UBE2T-induced G2/M arrest and DDR in HCC. (a)** UBE2T stably overexpression cells were treated with 2μM MK-8776 1h before 4 Gy IR, chromatin fraction was purified and analyzed for CHK1 by immunoblotting. **(b)** Cells were treated as that in panel a, fixed at the indicated timepoints after IR (4 Gy) and analyzed for cell cycle, the numbers of % cells in the G2 phase are shown. **(c)** Cells were treated as that in panel a, then were made for immunofluorescence staining of γH2AX. Representative images from triplicate experiments were shown, the quantification is shown in Fig.4e. Scale bar, 20μM. **(d)** Cells were treated as that in panel a, and analyzed for DNA damage by comet assay. Bar chart indicating the average tail moment per cell from triplicate experiments was shown in Fig. 4g.


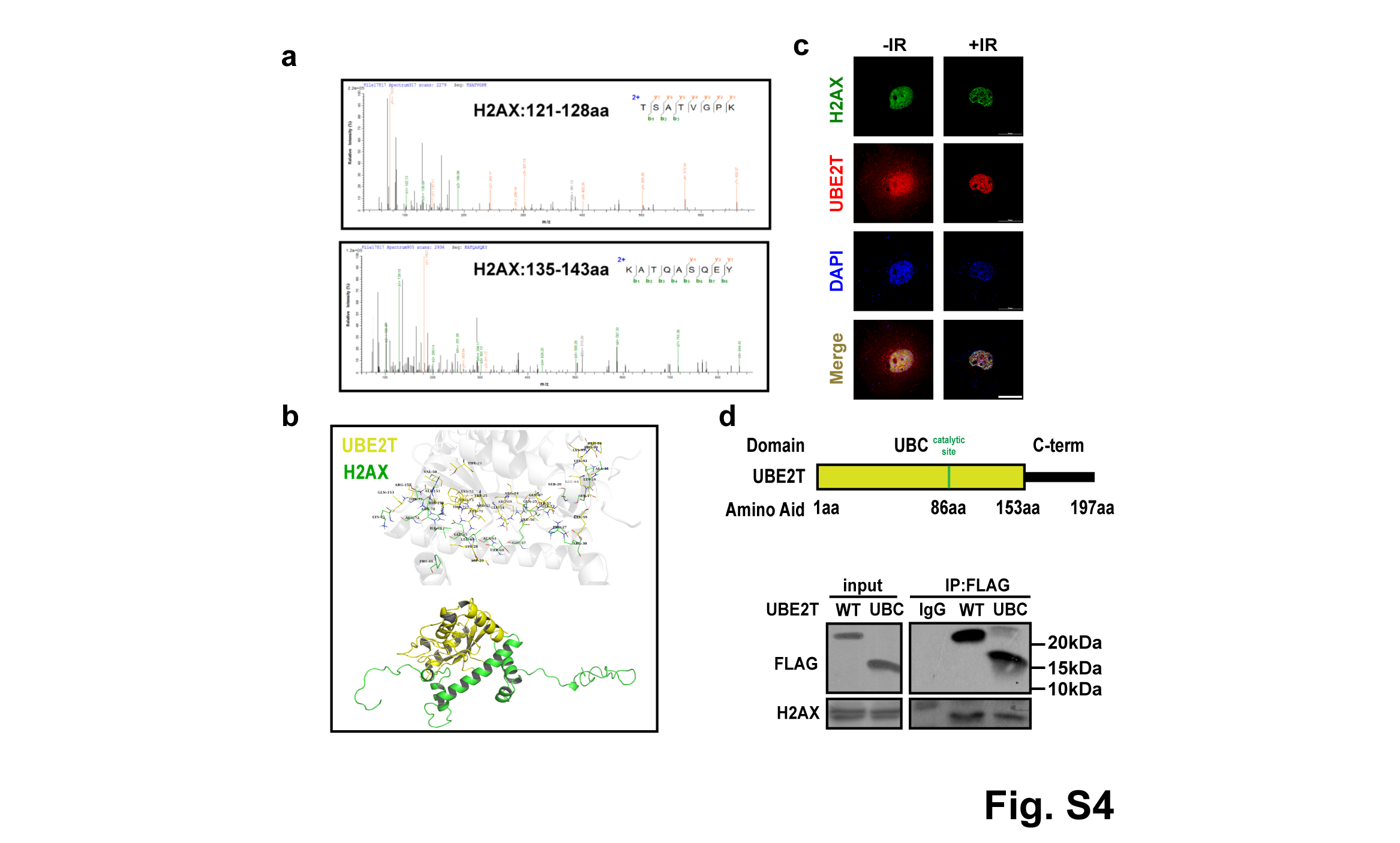


**Fig. S4 UBE2T binds with H2AX by UBC domain.** **(a)** H2AX was identified with sequence coverage using LC-MS/MS analysis. **(b)** The affinity and binding sites were predicted by molecular docking bioinformatic methods. **(c)** MHCC-97H cells transfected with FLAG-H2AX were exposed to IR (4 Gy) and stained with FLAG-H2AX (green) and UBE2T (red). **(d)** 293T cells were transfected with FLAG-UBE2T WT or FLAG-UBE2T UBC, and exposed to IR (4 Gy), immunoprecipitation was performed by using FLAG antibody, IP product was analyzed by immunoblotting.

**
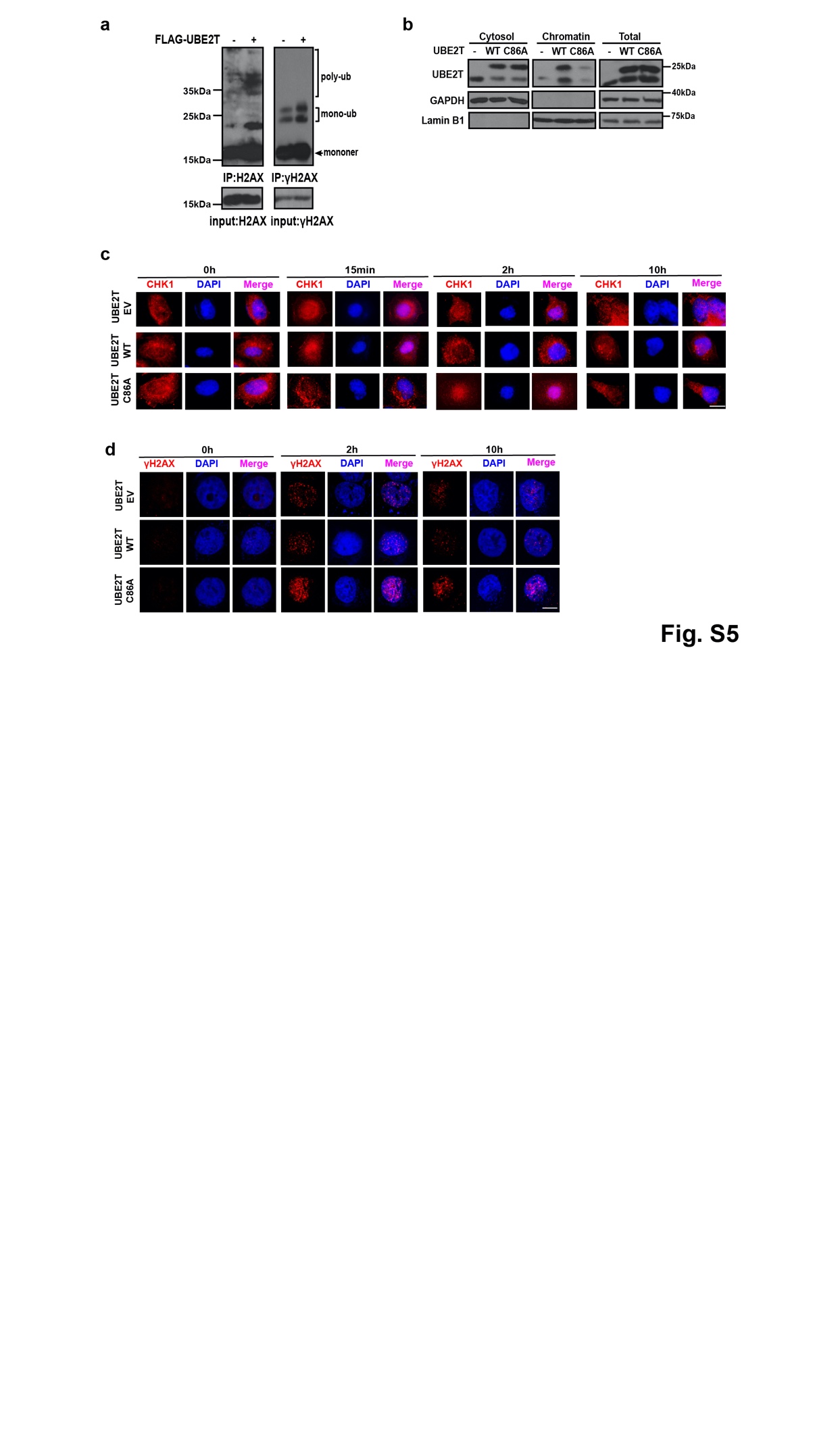
**

**Fig. S5 UBE2T C86A inhibits the chromatin accumulation of UBE2T, translocation of CHK1 from nuclear to cytosol and DDR after IR treatment. (a)** MHCC-97H cells were transfected by FLAG empty vector or FLAG-UBE2T, and treated with IR (4 Gy). Immunoblotting was performed to test H2AX/γH2AX ubiquitination. **(b)** MHCC-97H cells were transfected with FLAG-UBE2T WT or FLAG-UBE2T C86A, and treated with IR (4 Gy). Cytosol and chromatin fractionation was analyzed by immunoblotting. **(c-d)** MHCC-97H cells were transfected with adenoviral FLAG-UBE2T WT, FLAG-UBE2T C86A, or empty vector, treated with IR (4 Gy), harvested at indicated timepoints, and stained for CHK1 **(c)** and γH2AX **(d)**, representative images of immunofluorescence staining were shown. Scale bar, 20μM.

**
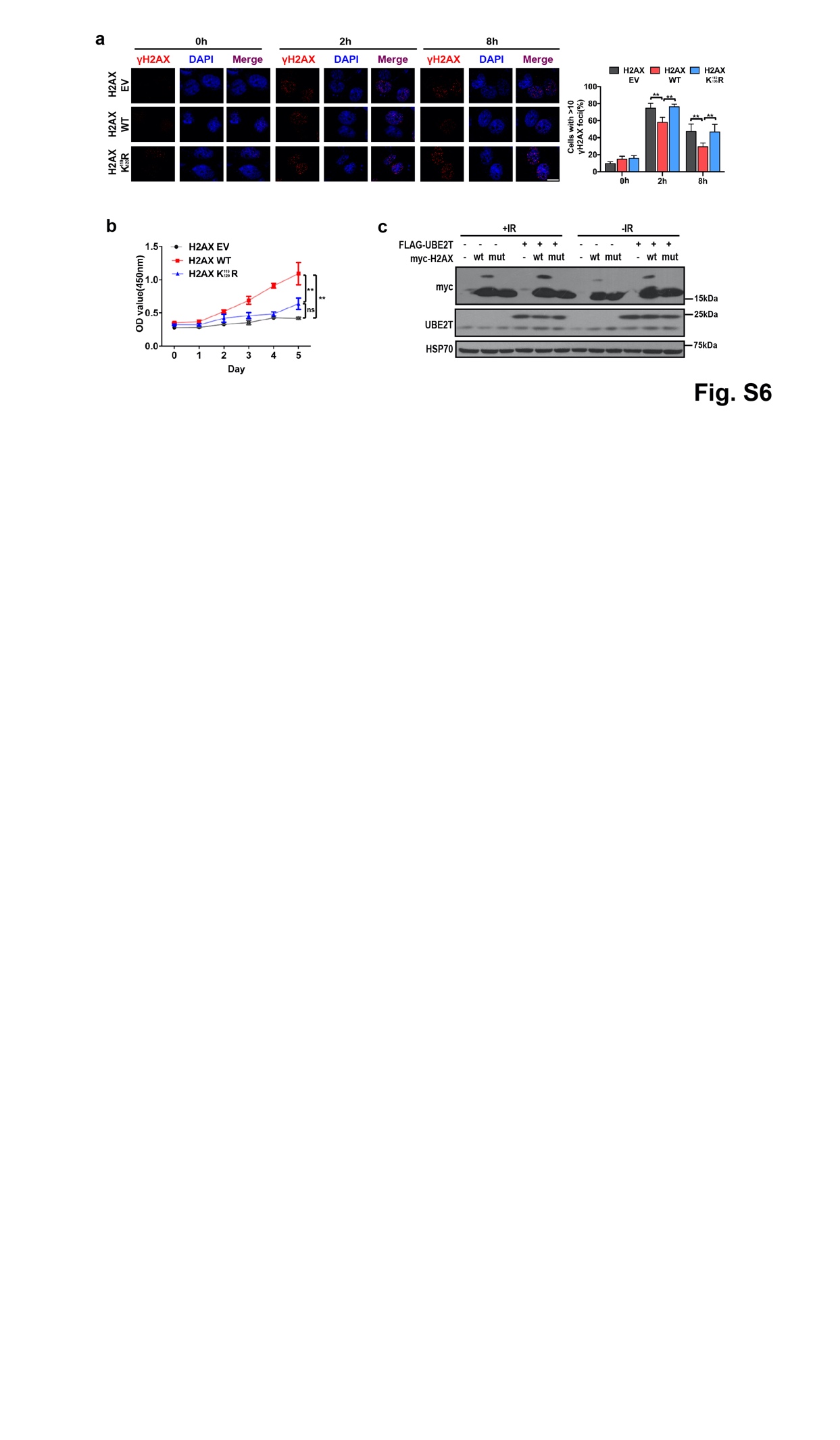
**

**Fig. S6 K119/120 are critical monoubiquitination sites and UBE2T regulates H2AX monoubiquitination after IR treatment in HCC cells. (a)** MHCC-97H cells were transfected with myc-tagged adenoviral-control, adenoviral-H2AX WT, or adenoviral-H2AX K119/120R, and stained for γH2AX at the indicated timepoints after IR (4 Gy). The representative images and quantification from triplicate experiments are shown. Scale bar, 20μM. Data represent the mean ± SD. ***P* < 0.01, by one-way ANOVA. **(b)** CCK-8 assays of the cells with the same treatment of panel a were shown. Data represent the mean ± SD from triplicate experiments. **P* < 0.05, by two-way ANOVA. (**c)** UBE2T overexpressing or control cells transfected with adenovirus-H2AX WT or adenovirus-H2AX K119/120R were exposed to IR (4 Gy). Total cell lysates were analyzed by immunoblotting.


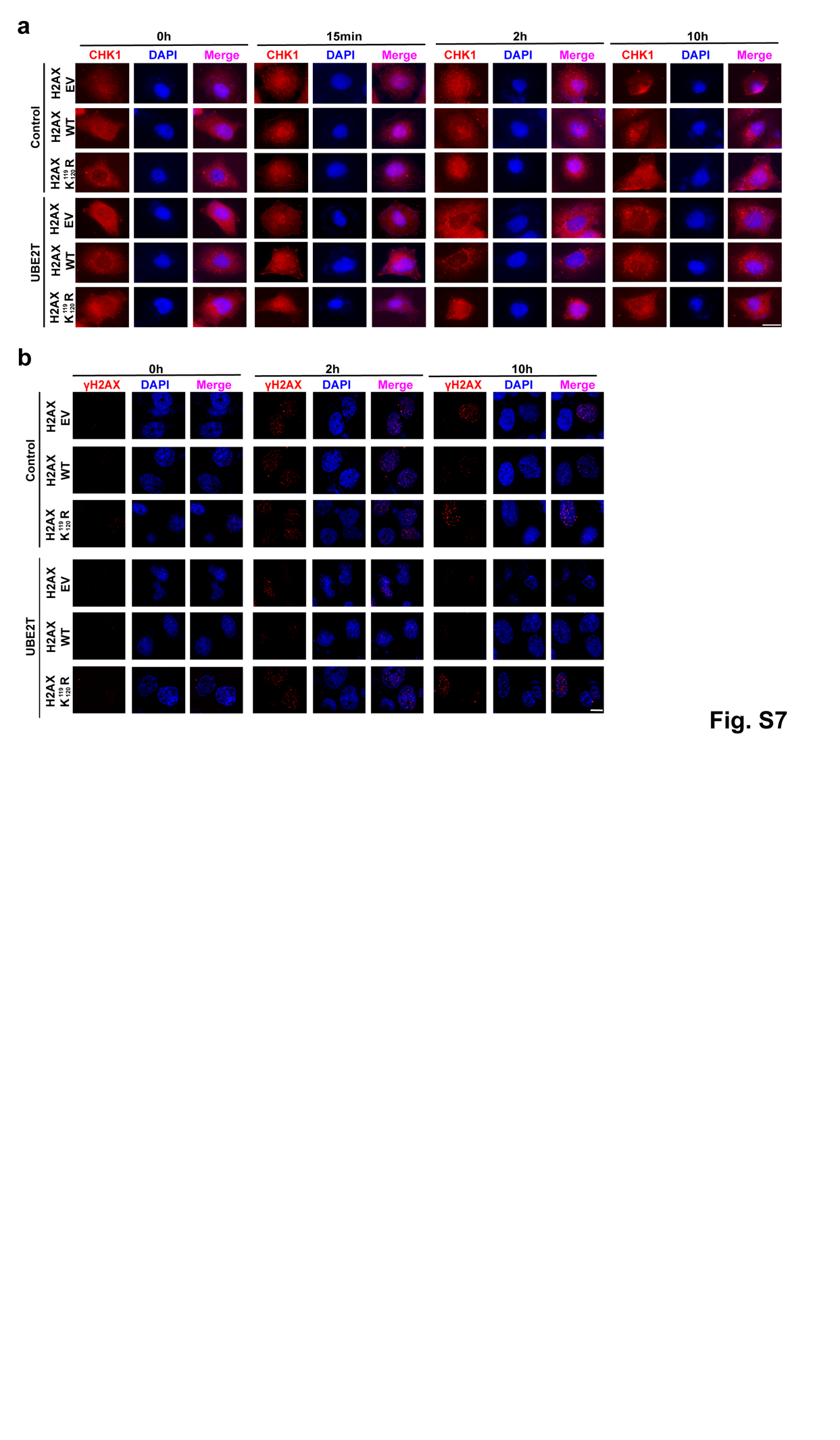


**Fig. S7 K119/120R impairs the role of UBE2T in promoting the translocation of CHK1 from nuclear to cytosol and DDR after IR treatment.** MHCC-97H cells were transfected with myc-tagged adenoviral-control, adenoviral-H2AX WT, adenoviral-H2AX K119/120R, or empty vector, treated with IR, harvested at indicated timepoints, and stained for CHK1 **(a)** and γH2AX **(b)**, representative images of immunofluorescence staining were shown.


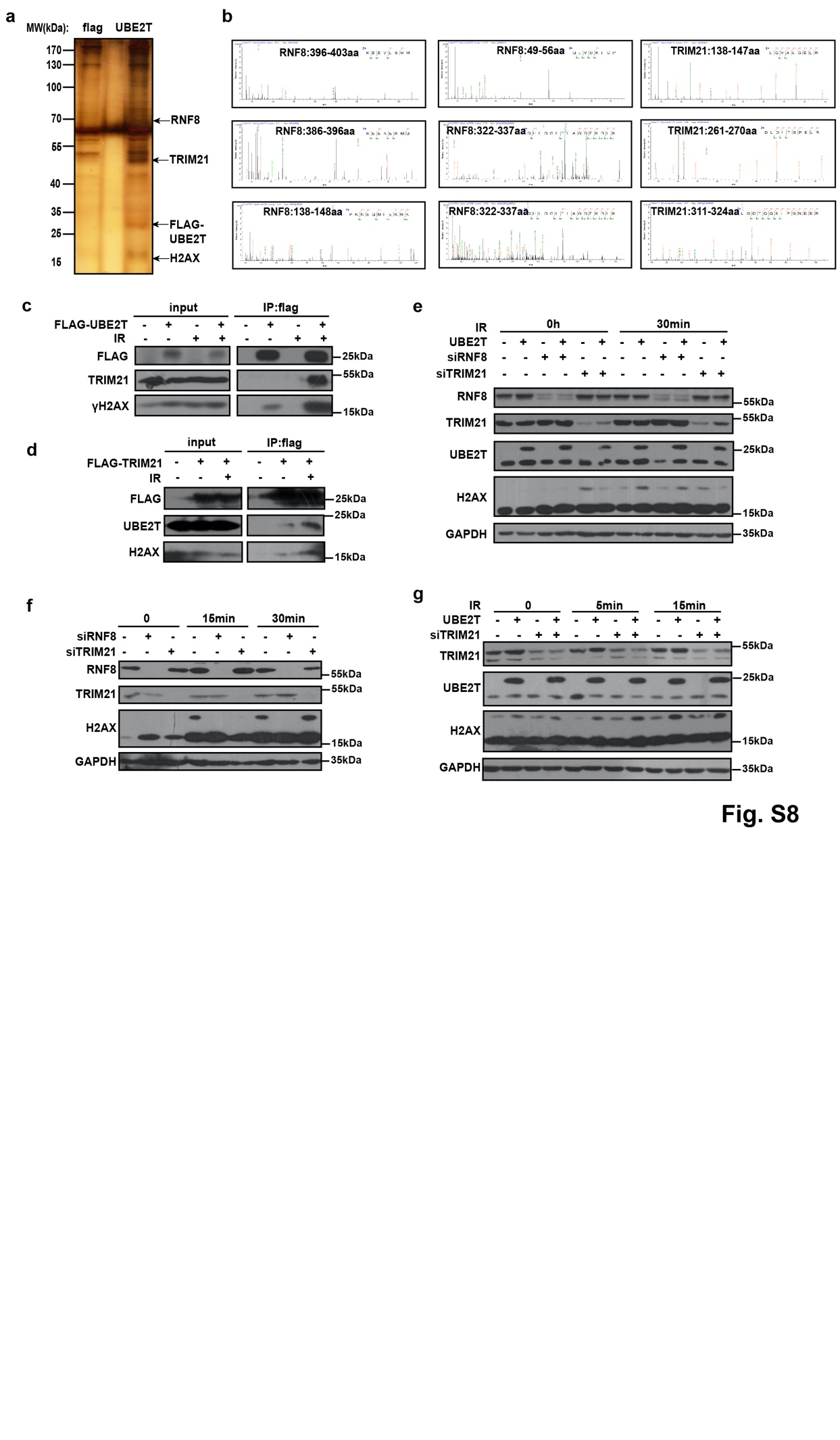


**Fig. S8 UBE2T regulates H2AX monoubiquitination together with RNF8, but not TRIM21, after IR in HCC cells.** **(a)** Silver-stained gel demonstrating an elution product after UBE2T immunoprecipitation in 293T cells transfected with empty vector or FLAG-UBE2T. H2AX, TRIM21 and RNF8 bands are indicated. **(b)** RNF8 and TRIM21 was identified with sequence coverage using MS analysis. **(c)** 293T cells were transfected with FLAG-UBE2T and exposed to IR (4 Gy), immunoprecipitation was performed by using FLAG antibody, IP product was analyzed by immunoblotting. **(d)** Immunoblotting analysis of FLAG-IP derived from the 293T cells after IR (4 Gy), which were transfected with empty vector or FLAG-TRIM21. **(e)** UBE2T overexpressing MHCC-97H cells were transfected with siRNA-control, siRNA-TRIM21 or siRNA-RNF8. Total lysates were analyzed for H2AX monoubiquitination at the indicated timepoints after IR (4 Gy). **(f)** MHCC-97H cells were transfected with siRNA-control, siRNA-TRIM21 or siRNA-RNF8. Total lysates were analyzed for H2AX monoubiquitination at the indicated timepoints after IR (4 Gy). **(g)** UBE2T-overexpressing cells were transfected with siRNA-TRIM21. Total lysates were analyzed for H2AX monoubiquitination at the indicated timepoints after IR (4 Gy).


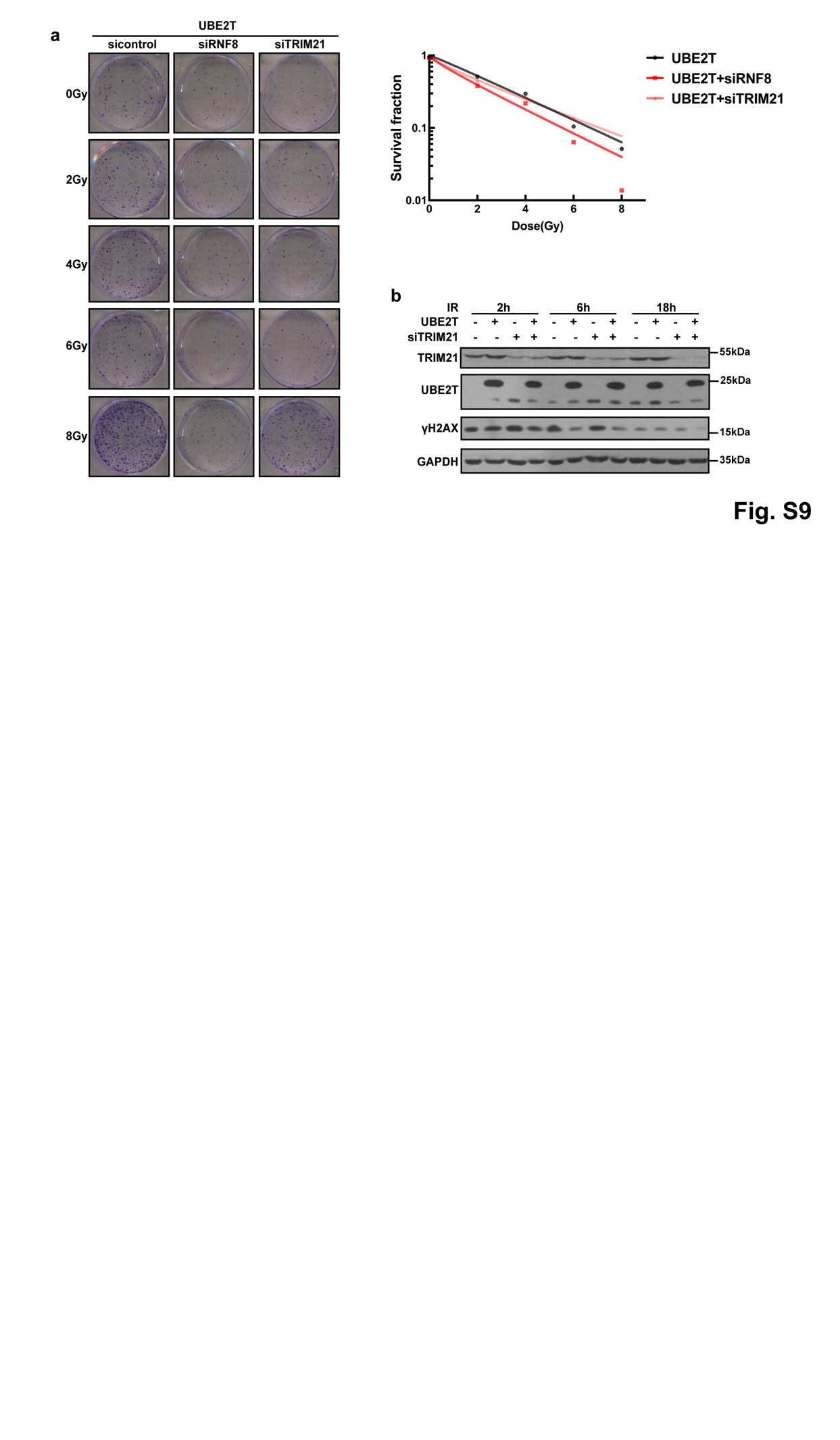


**Fig. S9 Knockdown of RNF8 but not TRIM21 reverts the effect of UBE2T on DDR and radioresistance.** **(a)** UBE2T overexpressing MHCC-97H cells were transfected with siRNA-control, siRNA-TRIM21 or siRNA-RNF8. Colony formation assay was conducted (left, representative images of colony formation from triplicate experiments; right, survival curve). **(b)** UBE2T-overexpressing MHCC-97H cells and control cells were transfected with siRNA-TRIM21. Total lysates were analyzed for γH2AX level at the indicated timepoints after IR (4 Gy).


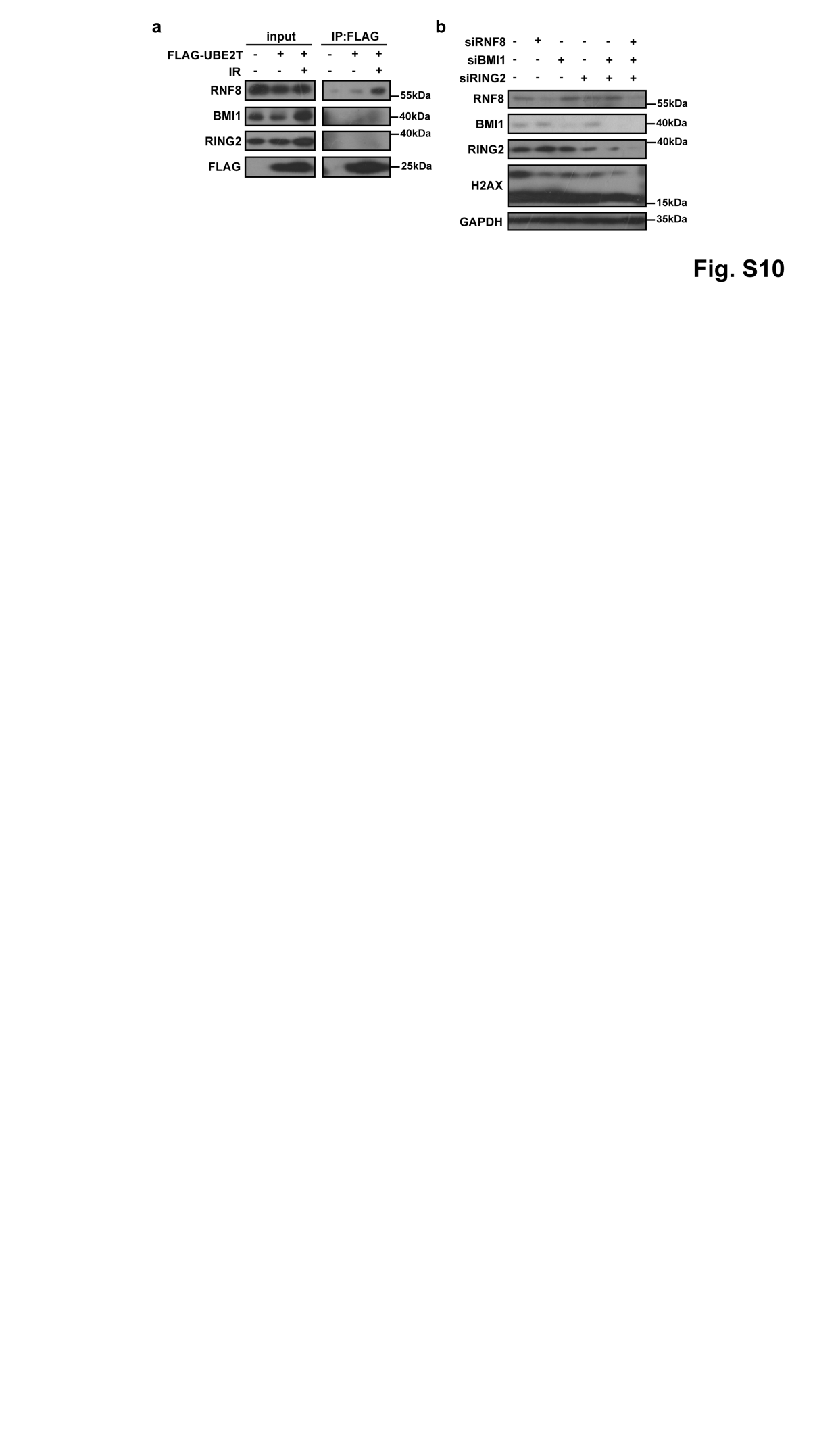


**Fig. S10 BMI1/RING2 don’t bind with UBE2T after IR exposure, and knockdown of RNF8 further decrease H2AX monoubiquitination upon knockdown of BMI1/RING2. (a)** 293T cells were transfected with FLAG-UBE2T and exposed to IR (4 Gy), immunoprecipitation was performed by using FLAG antibody, IP product was analyzed by immunoblotting. **(b)** MHCC-97H cells were transfected with the indicated siRNA, cells lysates were analyzed for H2AX monoubiquitination by immunoblotting.

**Table. S1 Clinicopathological characteristics of 133 HCC patients.**

| Feature N | | |  |  |
| --- | --- | --- | --- | --- |
| Gender  Male  Female | 113  20 | | |  |
| Age  ≤55  >55 | 80  53 | | |  |
| Edmondson Grade  Ⅰ+Ⅱ  Ⅲ+Ⅳ | 112  21 | | |  |
| Liver cirrhosis  With  without | 86  47 | | |  |
| HBV  Positive  negative | 107  26 | | |  |
| Envelop |  | | |  |
| With  Without | 85  48 | | |  |
| ALB(g/L）  ≤40  >40 | 75  58 | | |  |
| AST(U/L)  ≤40:  >40 | 65  68 | | |  |
| ALT(U/L)  ≤45  >45 | 73  60 | | |  |
| AFP (μg/L)  ≤20  >20 | 43  90 | | |  |
| Serum total bilirubin(μmol/L)  ≤17  >17 | | 85  58 | | |
| Tumor Size(cm) |  | | |  |
| ≤5  >5 | 43  90 | | |  |
| Relapse |  | | |  |
| Yes  No | 48  85 | | |  |
| Portal vein tumor thrombus  No  Yes | 96  37 | | |  |
| No. tumor  Solitary  Multiple | 95  38 | | |  |
| Metastasis |  | | |  |
| Yes  No | 15  118 | | |  |
| BCLC stage  A  B+C+D | 73  60 | | |  |

**Table. S2 Clinicopathological characteristics of 14 HCC patients received RT.**

| Characteristic | P1 | P2 | P3 | P4 | P5 | P6 | P7 | P8 | P9 | P10 | P11 | P12 | P13 | P14 |
| --- | --- | --- | --- | --- | --- | --- | --- | --- | --- | --- | --- | --- | --- | --- |
| Age(years) | 72 | 61 | 59 | 51 | 63 | 48 | 40 | 55 | 44 | 67 | 51 | 55 | 36 | 54 |
| Gender | M | M | M | M | M | M | M | M | F | M | M | M | M | M |
| AFP(ng/ml) | 4.3 | 304.7 | 0.7 | 2.2 | 11.7 | N/A | 12.3 | 413.3 | N/A | 5.7 | N/A | 2.6 | 149.7 | 20150.4 |
| ECOG | 1 | 1 | 1 | 0 | 1 | 0 | 1 | 1 | 2 | 2 | 1 | 2 | 1 | 1 |
| BCLC stage | C | B | C | A | C | C | C | C | C | C | A | C | A | C |
| Pathology | HCC | HCC | HCC | HCC | HCC | HCC | HCC | HCC | HCC | HCC | HCC | HCC | HCC | HCC |
| HBV | + | + | - | + | + | + | + | + | + | + | + | + | + | + |
| RT technique | IMRT | IMRT | IMRT | IMRT | IMRT | IMRT | IMRT | IMRT | IMRT | IMRT | IMRT | IMRT | IMRT | IMRT |
| RT sites | IHM | IHM | RMLN | IHM | STM | IHM | RMLN | STM | LM | STM | IHM | STM | IHM | RMLN |
| UBE2T score | 5 | 3 | 5 | 1 | 4 | 7 | 4 | 3 | 6 | 5 | 5 | 4 | 2 | 3 |

Abbreviations: IHM, Intrahepatic metastasis; LM, Lung metastasis; RMLN, Retroperitoneal metastatic lymph node; STM, Soft tissue metastasis; +，positive; -，negative.
